# Transmembrane water-flux through SLC4A11: a route defective in genetic corneal diseases

**DOI:** 10.1093/hmg/ddt307

**Published:** 2013-06-27

**Authors:** Gonzalo L. Vilas, Sampath K. Loganathan, Jun Liu, Andri K. Riau, James D. Young, Jodhbir S. Mehta, Eranga N. Vithana, Joseph R. Casey

**Affiliations:** 1 Membrane Protein Disease Research Group, Department of Biochemistry, University of Alberta, Edmonton, Alberta T6G 2H7, Canada; 2 Singapore Eye Research Institute, 11 Third Hospital Avenue, Singapore168751, Singapore; 3 Department of Ophthalmology, National University of Singapore, Singapore117597, Singapore; 4 Department of Clinical Sciences, Duke-NUS Graduate Medical School, Singapore169857, Singapore

## Abstract

Three genetic corneal dystrophies [congenital hereditary endothelial dystrophy type 2 (CHED2), Harboyan syndrome and Fuchs endothelial corneal dystrophy] arise from mutations of the *SLC4a11* gene, which cause blindness from fluid accumulation in the corneal stroma. Selective transmembrane water conductance controls cell size, renal fluid reabsorption and cell division. All known water-channelling proteins belong to the major intrinsic protein family, exemplified by aquaporins (AQPs). Here we identified SLC4A11, a member of the solute carrier family 4 of bicarbonate transporters*,* as an unexpected addition to known transmembrane water movement facilitators. The rate of osmotic-gradient driven cell-swelling was monitored in *Xenopus laevis* oocytes and HEK293 cells, expressing human AQP1, NIP5;1 (a water channel protein from plant), hCNT3 (a human nucleoside transporter) and human SLC4A11. hCNT3-expressing cells swelled no faster than control cells, whereas SLC4A11-mediated water permeation at a rate about half that of some AQP proteins. SLC4A11-mediated water movement was: (i) similar to some AQPs in rate; (ii) uncoupled from solute-flux; (iii) inhibited by stilbene disulfonates (classical SLC4 inhibitors); (iv) inactivated in one CHED2 mutant (R125H). Localization of AQP1 and SLC4A11 in human and murine corneal (apical and basolateral, respectively) suggests a cooperative role in mediating trans-endothelial water reabsorption. *Slc4a11^−/−^* mice manifest corneal oedema and distorted endothelial cells, consistent with loss of a water-flux. Observed water-flux through SLC4A11 extends the repertoire of known water movement pathways and call for a re-examination of explanations for water movement in human tissues.

## INTRODUCTION

Human cornea is arranged in five layers: the outer corneal epithelium, Bowman's layer that maintains the cornea's shape, the corneal stroma, composed mainly of tightly packed collagen fibrils secreted by keratocytes which occupy about 10% of the layer, the Descemet membrane, a modified basement membrane and the corneal endothelium, which is a monolayer of mitochondria-rich cells. High tissue swelling pressure in the corneal stroma arises as a result of collagen-related glcyosaminoglycans. Active transport-dependent mechanisms within the endothelial cell layer counter fluid accumulation by balanced water efflux (often called the ‘fluid pump’) through the endothelial cell layer into the aqueous humour, using incompletely characterized processes (1,2). Mutations in *SLC4A11* (also called NaBC1 or BTR1) (3,4) were recently identified in a range of corneal endothelial disorders characterized by dysfunction of the endothelial cells forming the inner surface of the cornea (5–7). These diseases are marked by fluid accumulation in the corneal stroma and abnormal deposition of material (gutatta) on the Descemet membrane that underlies the corneal endothelial layer (1).

Corneal dystrophies give rise to serious deterioration of vision from defects of the endothelial cell layer, which cause fluid accumulation and thickening of the stroma layer (1). Partial symptomatic relief can be achieved by dehydration of the cornea with hyper-osmotic saline eye drops, although full cure requires corneal transplantation. Mutations of *SLC4A11* cause some cases of the rare recessive diseases congenital hereditary endothelial dystrophy (CHED type 2, #217700) (5) and Harboyan syndrome (HS, OMIM #217400) (6). More significantly, SLC4A11 causes some cases of Fuchs endothelial corneal dystrophy (FECD, OMIM #613268), the most common cause of corneal transplant, accounting for up to 25% of corneal grafts (1). Among people over age 40, 4% develop this dominantly inherited, progressive disease. FECD is, however, genetically heterogeneous; two other genes have been reported as responsible for some early-onset FECD (8–10) and late-onset FECD is caused by mutations of *LOXHD1* (11). In addition to corneal disease, *SLC4A11* mutations also cause sensorineuronal deafness (Harboyan Syndrome) due to defects in the inner ear's stria vascularis, the structure responsible for maintenance of the composition of the inner ear's endolymph (6,12). More recently, hearing defects have been found in individuals with FECD (13). Disruption of murine *SLC4A11* gene recapitulates these human phenotypes (12,14), underscoring the conserved role of SLC4A11 in corneal and inner ear function.

SLC4A11 is an intriguing protein. Genetically, it is clearly a member of the SLC4 family of bicarbonate transporters (Supplementary Material, Fig. S1). Yet, both yeast and plant orthologues of SLC4A11 confer resistance to borate deficiency and function as borate transporters (15,16). Similarly, human SLC4A11 was reported to function as a sodium-coupled borate transporter (4). Borate has essential roles in bacteria, plants and fungi, where it cross-links vicinal diols to stabilize the structure of cell walls (17,18). The absence of a cell wall in mammalian cells, however, leaves no clear biochemical role for borate in mammals. Accordingly, borate transport by SLC4A11 does not provide an obvious explanation for the corneal diseases arising from *SLC4A11* mutations.

NIP5;1, a plant MIP protein (Supplementary Material, Fig. S1), functions as both a water and borate translocation pathway (19). This suggested to us that water movement and borate transport could require similar proteins. The intra-corneal fluid accumulation in individuals with SLC4A11 mutations led us to consider whether SLC4A11, functioning to facilitate water-flux, might better explain the corneal symptoms than defective borate transport. In keeping with such a potential role, SLC4A11 is abundantly expressed in the renal descending loop of Henle (12) where water reabsorption concentrates urine. Furthermore, *SLC4A11* null mice excrete dilute urine (12), consistent with a defect in water homeostasis.

Regulation of cell volume is a principal homeostatic mechanism in all cells. For example, during mitosis, cell volume clearly must expand to accommodate the need for a new cell's cytoplasm. In the kidney, urinary concentration requires water movement across the renal tubular epithelium. To date, all identified proteins that exclusively mediate water-flux across the plasma membrane belong to the major intrinsic protein (MIP) family, found throughout eukaryotes, but especially important in plants (20). In mammals, the only established water-flux pathway is formed by aquaporin proteins (AQPs), which themselves are part of the MIP family (20). The corneal fluid accumulation central to corneal dystrophies led us to consider whether the human protein, SLC4A11, functions as a previously unrecognized water movement pathway.

We examined the ability of human SLC4A11 to mediate water-flux, when expressed in *Xenopus laevis* oocytes and HEK293 cells. Characterization of the corneal phenotype of an *SLC4A11* null mouse strain that we prepared revealed that loss of SLC4A11 induces corneal oedema and progressive disorganization of the corneal endothelium. These data lead us to conclude that the function of SLC4A11 is to facilitate the movement of water across the basolateral corneal endothelium.

## RESULTS

### SLC4A11 facilitates transmembrane water-flux

**Figure 1.**
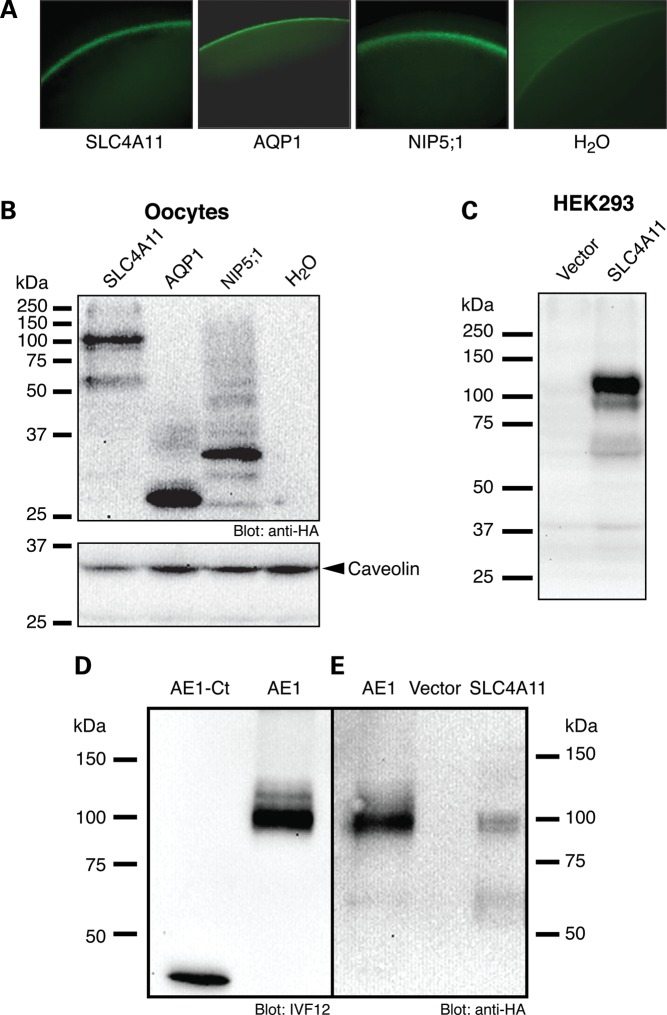
Quantification of SLC4A11 expression. (**A**) Confocal immunofluorescence of oocytes injected with the indicated RNA transcripts. HA-tagged proteins were detected using mouse anti-HA antibody and visualized using chicken anti-mouse IgG conjugated with Alexa Fluor 488 (green). (**B**) Oocytes, injected with cRNA encoding HA-epitope tagged SLC4A11, AQP1, NIP5;1 or with water alone were fractionated to produce a membrane-rich extract. Samples corresponding to 10 oocytes were probed on immunoblots with anti-HA antibody and caveolin antibody as a marker of the amount of membrane protein present, to enable measurement of the amount of HA-tagged protein/oocyte. (**C**) HEK293 cells were transfected with SLC4A11 cDNA or with vector alone were probed on immunoblots with a polyclonal antibody generated in rabbit against a synthetic peptide corresponding to amino acids 37–50 of human SLC4A11, in the putative cytoplasmic N-terminal domain of the protein. (**D**) AE1-Ct (40 ng protein) and 50 µg of protein from HEK293 cells transfected to express HA-epitope-tagged human AE1 (25) were immunoblotted and probed with mouse monoclonal anti-AE1 antibody, IVF12 (46). Densitometry of the immunoblot revealed the relative amounts of AE1 antigen present in the two preparations. (**E**) HEK293 cell lysate, containing 60 ng AE1, was electrophoresed along with a lysate of *X. laevis* oocyte membranes, corresponding to 10 oocytes. Densitometry revealed that each oocyte therefore contains 15 ng SLC4A11, corresponding to 9.1 × 10^9^ SLC4A11/oocyte.

**Figure 2.**
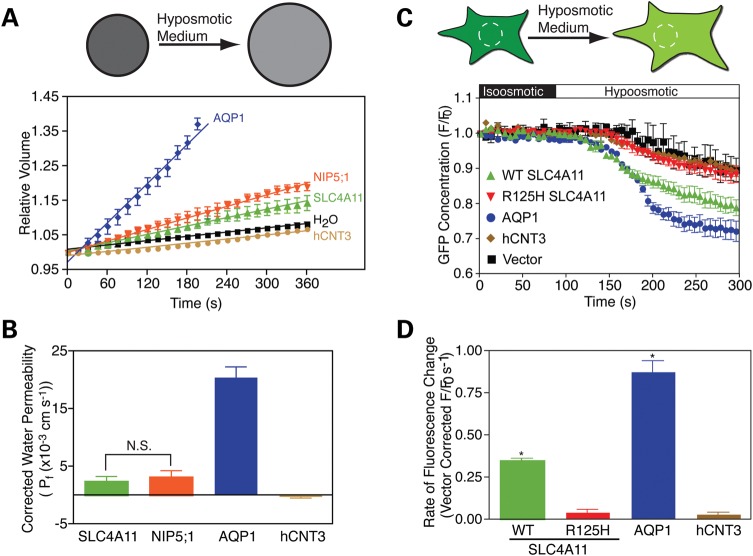
Water-flux through SLC4A11. (**A**) *Xenopus* oocytes were injected with H_2_O or H_2_O containing RNA transcripts encoding N-terminally HA-tagged SLC4A11, AQP1, NIP5;1 or untagged hCNT3. Osmotic swelling was induced by perfusing the oocytes with 44 mOsm/kg hypotonic solution at time zero, and oocyte volume was calculated from images captured digitally every 15 s. Top of panel diagrammatically shows a spherical oocyte swelling in response to hypo-osmotic medium. (**B**) Osmotic water permeabilities (P_f_) were calculated (23) for oocytes and corrected for the value observed in H_2_O-injected oocytes (2.9 ± 0.09 × 10^–3^ cm s^−1^). Bars represent mean ± SE from three separate swelling assays with 10 oocytes per assay. N.S., not significant. (**C**) HEK293 cells were transiently co-transfected with eGFP cDNA and SLC4A11 (WT and R125H mutant), hCNT3, AQP1 cDNAs or empty vector. Cells were perfused alternately with isotonic (black bar) and hypotonic (white bar) medium. eGFP fluorescence in regions of interest was measured digitally from images captured every 250 ms, but here only one data point every 3 s is shown. Top of panel diagrammatically represents a eGFP-expressing cell swelling in response to hypotonic medium. White circle represents the region of interest monitored to measure the change of eGFP concentration. (**D**) Rate of fluorescence change, calculated by linear regression of the initial intensity change during the first 15 s of perfusion with hypotonic buffer, was corrected for activity of vector-transfected cells (0.19 ± 0.005 s^−1^). Data represent the mean ± SE of six independent swelling experiments with 30–40 cells measured per assay. *Significant difference (*P* < 0.01) compared with hCNT3.

Water-flux through AQP proteins has been thoroughly studied and serves as a reference to study water movement by SLC4A11 (20,21). Most functional studies of AQP proteins monitor the increase of cell volume in AQP-expressing oocytes of *X. laevis*, following hypotonic challenge. SLC4A11, AQP1 and NIP5;1 were tagged at their N-termini with a hemagglutinin epitope tag to enable the proteins to be detected and quantified (Fig. 1). The human nucleoside transport protein, hCNT3 (22), was used as an additional control. Water-flux driven by an osmotic gradient, and uncoupled to solute transport, was induced by varying the concentration of membrane-impermeant solute, mannitol, without any other change of medium composition. Oocytes were incubated in iso-osmotic ND96 medium at 220 mOsmol/kg and shifted to the same medium at 44 mOsmol/kg (with less mannitol). Cell swelling was assessed by measuring changes of oocyte diameter as a function of time. AQP1-expressing oocytes rapidly increased in volume, to the extent that they burst (Fig. 2A), as found in the original studies of AQP1 (21). SLC4A11-expressing oocytes swelled more rapidly than water-injected or hCNT3-expressing oocytes (Fig. 2A). Water-permeability values (P_f_) (23) were measured for oocytes expressing the different proteins (Fig. 2B). The indistinguishable water permeability found in water-injected and hCNT3-expressing oocytes indicates that the ability to confer water permeation is not a general feature of membrane transport proteins.

Since proteins are expressed to different levels, we examined the abundance of SLC4A11, AQP1 and NIP5;1 in oocyte membranes. Immunofluorescence of oocytes revealed similar plasma membrane localization of SLC4A11, AQP1 and NIP5;1 (Fig. 1A), but this technique is not quantitative. Immunoblots of oocyte membrane lysates revealed the relative abundance of these proteins, which were all tagged with the HA-epitope (Fig. 1B). In order to calculate unitary water permeation rates, we needed to establish the absolute number of protein molecules present in oocyte membranes. To do so, we made use of a GST-fusion protein corresponding to the C-terminal 40 amino acids of human AE1, fused to Glutathione-S-Transferase, which was called AE1-Ct (24). This fusion protein was purified to homogeneity and its abundance quantified by protein assay, allowing it to serve as a standard for antigen abundance. An immunoblot containing AE1-Ct and full length AE1 (tagged with HA in an extracellular loop) (25) was probed with a monoclonal antibody that recognizes an epitope in the C-terminus of AE1, allowing standardization of molar abundance of full length AE1 in the lysate (Fig. 1D). The same cell lysate containing the HA-tagged AE1 was run on another immunoblot, along with lysate from oocytes expressing HA-tagged SLC4A11 (Fig. 1E). Probing the immunoblot with an anti-HA antibody enabled quantification of the molar abundance of SLC4A11 in the oocyte lysate.

We then determined the unitary water-flux carried by proteins expressed in oocytes. With the knowledge of the concentration of SLC4A11, we were able to calculate the concentration of HA-tagged proteins in membrane lysates (Fig. 1B). From the rate of oocyte diameter change following switch to hypotonic medium, we calculated the volume of water.s^−1^, which was readily converted into molecules H_2_O s^−1^. Taken together, we established the rates of water movement through each protein molecule as 5 ± 2 × 10^7^ H_2_O s^−1^ and 0.9 ± 0.3 × 10^9^ H_2_O s^−1^ for SLC4A11 and AQP1, respectively. The value for AQP1 aligns well with its reported water-flux (10^9^ H_2_O s^−1^) (26). Since the water-flux through AQP0 is only 10^8^ H_2_O s^−1^ (26), SLC4A11 mediates water-flux in the range of some AQP proteins.

To examine water movement by SLC4A11 in the setting of a mammalian cell, we performed additional experiments in transfected HEK293 cells (Fig. 2C and D). HEK293 cells were co-transfected with cytosolic enhanced green fluorescent protein (eGFP) and SLC4A11, AQP1, hCNT3 or vector alone. Using confocal microscopy, the cytosolic eGFP concentration was monitored as cells were exposed to iso-osmotic medium and shifted to a hypotonic medium of identical composition, except for having a lower concentration of the membrane-impermeant compound, mannitol. The hypotonic challenge causes cell swelling, which manifests as a dilution of cytosolic eGFP, measured by image analysis of a small region of interest in the cytosol (Fig. 2C, upper). The cytosolic concentration of eGFP is thus inversely proportional to cell volume and the rate of change of eGFP concentration provides a surrogate for the rate of volume change (Fig. 2C). Fluorescence of eGFP variants can be quenched by protons (27). If the hypo-osmotic medium induces a cytosolic acidification, it would be interpreted in our assay as a cell-swelling event. To examine whether this occurred, we transfected HEK293 cells with vector alone, hCNT3, AQP1 or SLC4A11, loaded them with the pH-sensitive dye, BCECF-AM and monitored changes in intracellular pH during perfusion with iso- and hypo-osmotic solutions (Supplementary Material, Fig. S2). Perfusion with hypotonic media resulted in a small but measurable intracellular acidification. However, there was no statistically significant difference between the acidification rates determined for cells expressing any of the proteins.

In HEK293 cells expressing hCNT3, volume changed at a rate indistinguishable from vector-control cells (Fig. 2D). SLC4A11-expressing cells, however, had a rate of volume change about half that of AQP1 (Fig. 2D). These rates do not account for possible differences in protein abundance (which was not possible to assess as the proteins do not share an epitope). We did, however, measure the efficiency of protein trafficking to the cell surface, revealing that 92 ± 1, 57 ± 3 and 44 ± 5% of hCNT3, SLC4A11 and AQP1, respectively,  processed to the cell surface when expressed in HEK293 cells (Supplementary Material, Fig. S3). While AQP1 processing to the cell surface was less than SLC4A11, it does not affect the conclusion that SLC4A11 induces a water-flux in HEK293 cells that is comparable to AQP1. Together, observations of oocyte and mammalian cell swelling establish the ability of SLC4A11 to facilitate water-flux without coupled solute transport.

### Mutations of SLC4A11 impair water-flux

We considered the possibility that SLC4A11 might not directly act as a water flow pathway, but could activate or unmask some water movement pathway endogenously found in cells. We explored this possibility, using a unique CHED-causing SLC4A11 mutation (R125H). Unlike other disease alleles of *SLC4A11*, which cause the protein to be retained in the endoplasmic reticulum, as a result of misfolding (5,7,28), R125H is transported to the cell surface just as wild-type (WT) (29). This suggests that the mutation causes disease by compromising function, rather than impairing protein localization to the cell surface. Indeed, R125H SLC4A11 does not facilitate water-flux when expressed in HEK293 cells (Fig. 2C and D), indicating that CHED arises when SLC4A11 is unable to facilitate water-flux across the plasma membrane.

**Figure 3.**
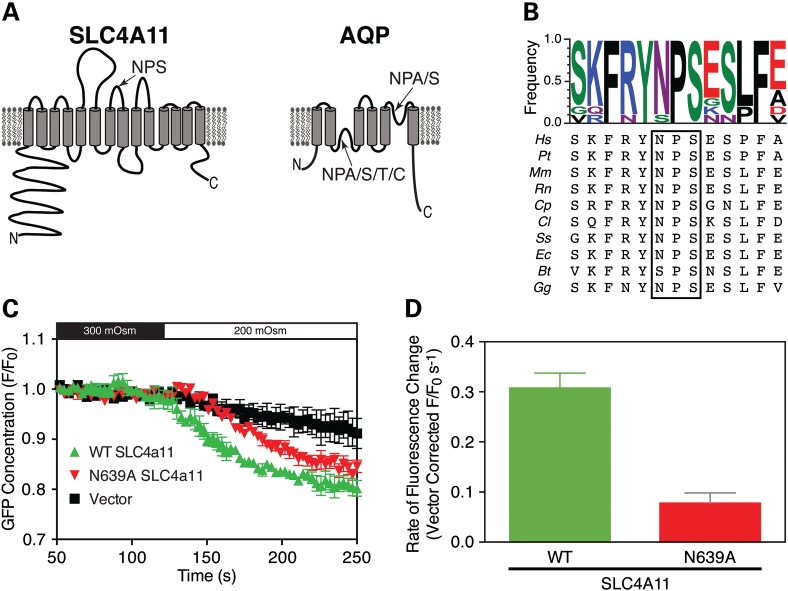
Loss of water-flux upon mutation of an NPS motif. (**A**) Schematic topology models for SLC4A11 and for the AQP family of proteins, with the outside of the cell at the top. Positions of conserved Asparagine–Proline-X (NPX) motifs are indicated by arrows. (**B**) Amino acid sequence alignments of the region around the NPX motif found in SLC4A11 (boxed). Species are: Human (*Homo sapiens* (*Hs*)), Chimpanzee (*Pan troglodytes* (*PT*)), Mouse (*Mus Musculus*, *Mm*), Rat (*Rattus norvegicus*, *Rn*), Guinea pig (*Cavia porcellus*, *Cp*), Dog (*Canis lupus familiaris, Cl*), Pig (*Sus Scrofa, Ss*), Horse (*Equss caballus*, *Ec*), Cattle (*Bos Taurus*, *Bt*) and Chicken (*Gallus gallus*, *Gg*). The degree of conservation is indicated at the top by amino acid frequency, represented by the relative height of each single-letter coded amino acid (WebLogo software) (47). (**C**) Water permeability of HEK293 cells expressing WT or N639A SLC4A11 was assessed, by following the rate of change of eGFP fluorescence after cells were exposed to hypotonic medium. (**D**) Comparison of WT and N639A rates of cell swelling following hypo-osmotic challenge. The rate of fluorescence change was corrected for activity of vector-transfected cells (0.19 ± 0.02 s^−1^). Data represent mean ± SE of three independent swelling experiments with 30–40 cells measured per assay.

The high-resolution structure for AQP1 has been instrumental in understanding the mechanism of water movement through the protein (30). In AQP proteins, conserved NPX (where x is Ala, Ser, Thr or Cys) motifs present in re-entrant loops are critical for water conductance and ion exclusion (Fig. 3A) (26), leading us to examine the role of NPX sequences in SLC4A11. In human SLC4A11, there is only one NPX sequence in the membrane domain (N639PS), which is highly conserved across species (Fig. 3B). When expressed in HEK293 cells, N639PS-A639PS SLC4A11 matured and trafficked normally to the cell surface (Supplementary Material, Fig. S4), but conferred 66% less water permeability than WT SLC4A11 (Fig. 3C). From this experiment we cannot, however, conclude that AQP proteins and SLC4A11 both use NPX motifs in the same way in their water conduction mechanism.

The reduced water-flux associated with A639PS SLC4A11 does, however, suggest that SLC4A11 directly forms a water-conductive route, rather than increasing water-flux through an endogenous pathway. The same conclusion arises from the lack of water-flux through R125H-SLC4A11.

### Dependence of water-flux on abundance of functional SLC4A11

**Figure 4.**
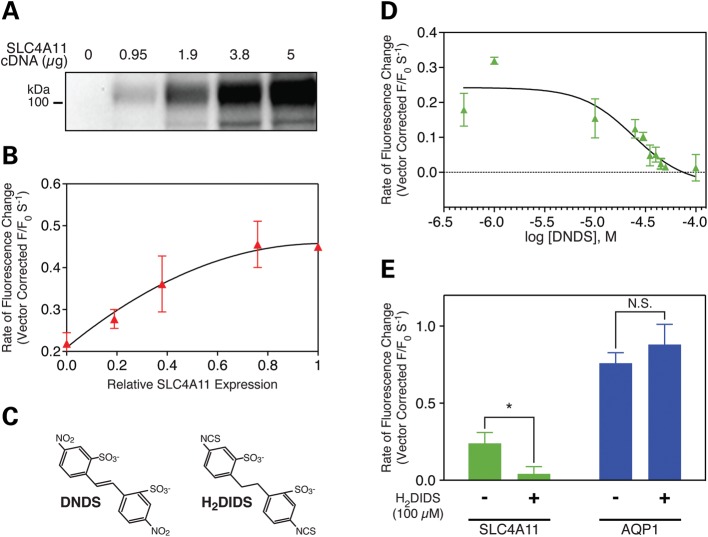
Water-flux is proportional to SLC4A11 expression level and is inhibited by stilbene disulfonates. (**A** and **B**) HEK293 cells were co-transfected with eGFP cDNA and indicated amount of cDNA encoding HA-tagged wild-type (WT) SLC4A11. (A) SLC4A11 expression was quantified on immunoblots of cell lysates probed with anti-HA antibody. (B) Cell swelling of HEK293 cells, expressing various amounts of SLC4A11, was assessed by the eGFP dilution assay (as in Fig. 2C). The rate of swelling observed for vector-alone transfected cells was subtracted from the values observed for cells expressing SLC4A11. (**C**) Chemical structures of SLC4A11 inhibitors used here. (**D** and **E**) HEK293 cells were co-transfected with eGFP and either SLC4A11, AQP1 or vector cDNA. Water-flux following shift to hypo-osmotic medium, assessed by measuring the rate of eGFP fluorescence change (as in Fig. 2C), was corrected for rate in vector-transfected cells. (D) Water-flux assays were performed in the presence of varied concentrations of DNDS, a non-covalently acting compound classically used to inhibit proteins of the SLC4 family (48). Half-maximal effective concentration determined from the curve is 24 ± 1 µM DNDS, *n* = 3. (E) eGFP dilution water-flux assays were performed in the absence or presence of 100 µM H_2_DIDS, a covalently acting stilbene disulfonate. Rates of fluorescence change were corrected for the value found for vector-transfected cells. *P < 0.05. N.S., not significant. *n* = 3.

We further explored the dependence of transmembrane water-flux on SLC4A11. In HEK293 cells expressing SLC4A11, the magnitude of water-flux was directly proportional to the expression level of SLC4A11 (Fig. 4A and B), arguing that SLC4A11 directly mediates water-flux. In a second experiment, we explored the ability to inhibit water-flux pharmacologically. Transport activity of Na^+^-dependent and independent bicarbonate transporters in the SLC4 family is inhibited by stilbene disulfonate compounds (Fig. 4C). SLC4A11 binds these compounds (29), but the effect on SLC4A11 function has not been previously assessed. The stilbene disulfonate compound, DNDS (Fig. 4C), inhibited water movement in SLC4A11 expressing HEK293 cells with half-maximal efficiency of 24 ± 1 µM (Fig. 4D). Moreover, 100 µM H_2_DIDS (Fig. 4C) blocked water-flux associated with SLC4A11, but did not affect flux through AQP1 (Fig. 4E), indicating that pharmacological inhibition of water-flux through SLC4A11 and AQP1 differs. Stilbene disulfonates bind SLC4A11 protein with half maximal concentration of 23 ± 7 µM (29), indicating that inhibitor binding and water-flux activities of SLC4A11 share sensitivity to stilbene disulfonates.

### Does SLC4A11 mediate Na^+^ accumulation?

Up to this point, the data indicate that expression of SLC4A11 induces water accumulation in response to hypo-osmotic challenge. An integral membrane protein, like SLC4A11, can induce water accumulation in two ways: (i) the protein can form a pathway that facilitates water movement (as in aquaporins) or (ii) the protein can induce solute entry into the cell, which drives parallel osmotic water accumulation. In the experiments here, we observed water accumulation induced by reduction of medium osmolarity, without change of concentration of *any* solute, except for membrane-impermeant mannitol. The simplest explanation for the observation, is mechanism 1 indicated earlier.

In these experiments, reduction of mannitol concentration provides an osmotic driving force, without changing the driving force for accumulation of any substrate. Therefore, if SLC4A11 causes increased solute accumulation in the hypo-osmotic medium, it must arise from accumulation of a solute for which there is a driving force for accumulation under iso- and hypo-osmotic conditions.

If mechanism 2 occurs, the likely candidate solute would be Na^+^, since the electrochemical gradient for this abundant solute is inward, many concentrative transporters are coupled to this ion and SLC4A11 was reported to have Na^+^ conductive activity (4). To test whether Na^+^ influx contributes to the rate of cell swelling induced by hypo-osmotic medium, we performed cell swelling assays in HEK293 cells in the presence and absence of Na^+^, where NaHCO_3_^−^ and NaCl in Na^+^-free media were replaced by KHCO_3_^−^ and choline chloride, respectively (Supplementary Material, Fig. S5). Since HEK293 cells endogenously express Na^+^/H^+^ exchange (NHE) activity, it was necessary to supplement media with the NHE inhibitor, EIPA, to prevent cellular acidification induced by NHE reversal under Na^+^-free conditions.

In Na^+^-containing medium, the presence of EIPA did not affect the rate of cell swelling in SLC4A11 expressing HEK293 cells (Supplementary Material, Fig. S5D). The rate of swelling was corrected for the rate observed for vector-transfected cells under each condition. Most significantly, the rate of swelling was indistinguishable in the presence and absence of Na^+^. The mechanism by which SLC4A11 induces an increased rate of swelling following hypo-osmotic challenge is Na^+^-independent.

### *Slc4a11*^−/−^ mice manifest corneal defects

**Figure 5.**
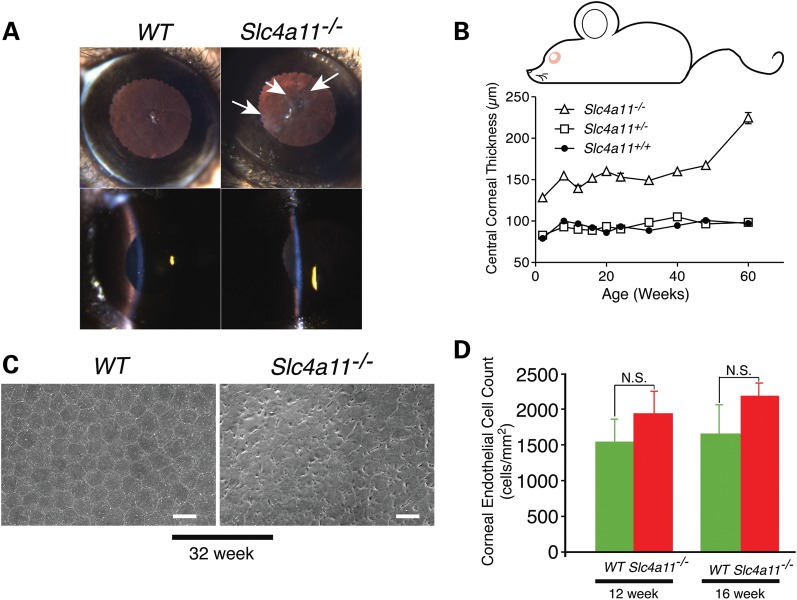
Corneal oedema and progressive corneal dysfunction in *slc4A-a11*^−/−^ mice. (**A**) Slit lamp examination of *WT* and *slc4a11*^−/−^ mice. Corneas of 48-week *WT* and *slc4a11*^−/−^ mice seen under retro-illumination (top panels) and examined using tangential slit illumination (bottom panels). Arrows indicate corneal haze observed in *slc4a11*^−/−^ mice. (**B**) Corneal thickness measured by *in vivo* confocal microscopy (*n* = 5 per genotype). Central corneal thickness was significantly different between *WT* and *slc4a11^−/−^* mice at all time points (*P* < 0.01). The corneal thickness in heterozygous (*slc4a11^+/−^*) mice was not different compared with wild-type mice. (**C**) Scanning electron micrographs of corneal endothelia from *WT* and *slc4a11^−/−^*mice at 32 weeks. Scale bars are 20 µm. (**D**) Statistical analysis of corneal endothelial cell density.

To characterize the role of SLC4A11 and its function *in vivo,* we developed a line of mice with a Cre-induced deletion of their *slc4a11* gene (Supplementary Material, Figs S6 and S7). Slit lamp examination revealed that *slc4a11*^−/−^ mice have hazy corneas (the cloudy appearance is indicated by arrows), indicating corneal oedema, whereas corneas of *WT* mice appeared normal and translucent (Fig. 5A). Consistent with these microscopic changes, *slc4a11*^−/−^ mice exhibit macroscopic corneal oedema and a progressive increase in corneal thickness (Fig. 5A and B). During aging, differences in endothelial cell morphology became evident (Supplementary Material, Fig. S8). Endothelial cells of 60-week-old *WT* mouse maintained polygonal shape, but were larger than those in the 12-week-old mouse. Swollen large endothelial cells were predominant in 60-week-old *slc4a11*^−/−^ mice with indiscernible cell boundaries (Supplementary Material, Fig. S8). In contrast to *WT* littermates, the corneal endothelium of *slc4a11^−/−^* mice displays progressive and profound disorganization over 12–60 weeks (Supplementary Material, Fig. S8). Endothelial cells of the *slc4a11*^−/−^ mice show membrane ruffling suggestive of cell swelling at 32 weeks (Fig. 5C). Endothelial cell density quantified at 12 and 16 weeks was not, however, different between *WT* and *slc4a11^−/−^* mice, indicating that loss of SLC4A11 does not affect cell proliferation (Fig. 5D) at an early stage in the disease. The progressive nature of the endothelial changes in this mouse model is similar and recapitulates the key aspects of human SLC4A11-associated diseases.

### Localization of water movement apparatus in the corneal endothelium

**Figure 6.**
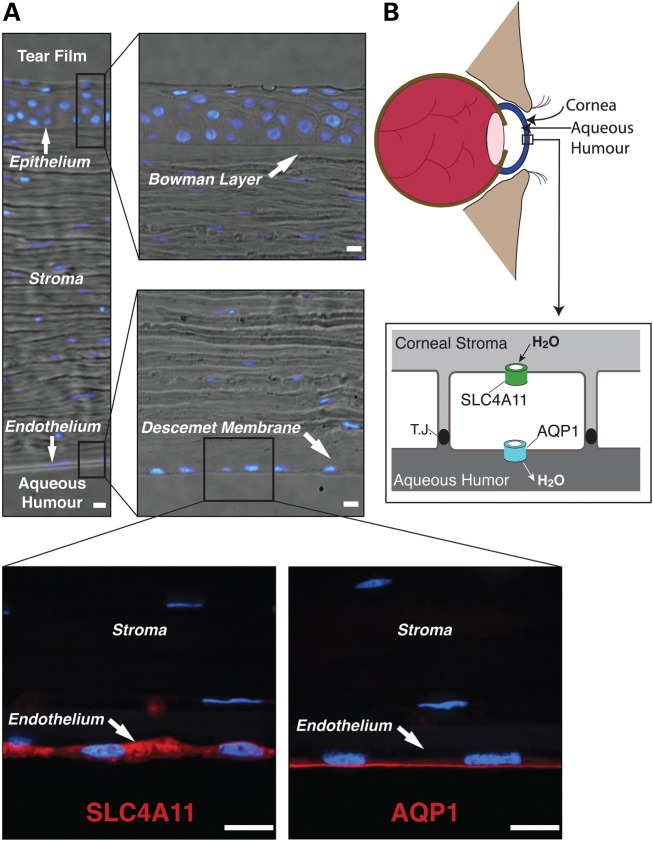
SLC4A11 and AQP1 in human cornea. (**A**) Bright field and confocal immunofluorescence microscopy images of paraffin-embedded human cornea. Cornea sections were incubated with rabbit anti-SLC4A11 or rabbit anti-AQP1 serum, followed by goat anti-rabbit IgG conjugated with Alexa Fluor 594 (red). Nuclei were detected with DAPI (blue). Scale bars represent 10 µm. (**B**) The endothelial cell layer separates the corneal stroma from the aqueous humour. Fluid accumulates in the stroma, which is reabsorbed by the endothelial cell layer to prevent corneal oedema. Localization of AQP1 and SLC4A11 to opposite poles of endothelial cells suggests they may work cooperatively to provide a water conductive pathway through the endothelial layer. T.J. represents the tight junction that connects cells of the endothelium.

Together these data suggest that loss of water movement capacity contributes to the corneal dystrophies and hearing defects in people with defective *SLC4A11*. Fluid accumulation in the corneal stroma arises from processes that either increase water accumulation in the stroma or decrease water reabsorption into the aqueous humour, which is accomplished by the corneal endothelial cell layer. Corneal endothelial cells are organized with the apical surface facing the aqueous humour and the basolateral surface toward the stroma. Immunolocalization of AQP1 and SLC4A11 in human cornea sections reveals AQP1 exclusively on the apical endothelial surface, while SLC4A11 is present on the opposite pole of the endothelial cells, along the basal and lateral surfaces (Fig. 6). Specificity of SLC4A11 localization is indicated by the absence of SLC4A11 fluorescent signal in sections incubated with non-immune serum instead of anti-SLC4A11 or anti-AQP1 (Supplementary Material, Fig. S9). AQP1 has been reported on both apical and basolateral surfaces of rat and human corneal endothelial cells (31,32), but our data suggest an exclusively apical localization for human AQP1. Examination of AQP1 and SLC4A11 in *WT* and *slc4a11^−/−^* mouse corneal sections revealed the same pattern of localization as in human cornea (Supplementary Material, Fig. S10). Importantly, SLC4A11 immunoreactivity was absent from the *slc4a11^−/−^* corneal sections (Supplementary Material, Fig. S10), indicating specificity of immunolocalization. Localization of SLC4A11 in mouse cornea provides additional support for basolateral localization of the protein, as it co-localized with the basolateral markers, NHE1 (sodium/proton exchanger) and the Na^+^/K^+^-ATPase (Supplementary Material, Fig. S11).

Failed water reabsorption in genetic corneal dystrophies can be explained by loss of water-flux across the basolateral endothelial surface. While AQP1 will facilitate water movement across the apical surface, fluid reabsorption across the cell layer requires a water-conductive pathway across the basolateral surface (Fig. 6B). The functional activity and localization reported here suggest that SLC4A11 provides a pathway for water-flux through the basolateral membrane, which is required for reabsorption of fluid from the corneal stroma.

## DISCUSSION

These studies examined the molecular defect present in individuals whose corneal disease arose from mutations of *SLC4A11*. Our data show that SLC4A11 protein forms a previously unrecognized water-conductive pathway, the first such route identified in a protein that is not part of the MIP family. Data here provide an explanation for the corneal diseases caused by SLC4A11 defects. Expression of SLC4A11 disease-causing mutants in mammalian cell lines revealed that the mutations caused retention of the protein in the endoplasmic reticulum (5,7). Assayed in *X. laevis* oocytes and HEK293 cells, SLC4A11 mediated water-flux driven exclusively by an osmotic gradient. Our data are consistent with a role for SLC4A11 in forming a basolateral trans-endothelial cell water pathway required for fluid reabsorption from the corneal stroma, working in concert with apical AQP proteins (Fig. 6B).

### SLC4A11 mediates transmembrane water-flux

Our data support a direct role of SLC4A11 in mediating the movement of water across membranes. SLC4A11-expressing oocytes had an increased rate of cell swelling when incubated in hypo-osmotic medium that had the same composition as the iso-osmotic medium, except for reduced concentration of membrane-impermeant mannitol. The only driving force for water movement was therefore osmotic, since the chemical composition of the iso- and hypo-osmotic solutions was the same. In HEK293 cells, SLC4A11-expressing cells swelled when exposed to hypo-osmotic medium at a rate about one-third of AQP1-expressing cells again indicating that SLC4A11-expressing cells respond in a manner similar to cells expressing the bona fide water channel, AQP1.

SLC4A11 could act also by activating an endogenous water movement pathway, but the data argue against this possibility. Increased osmotically driven water movement was observed in SLC4A11-expressing oocytes and HEK293 cells, which are unlikely to express the same endogenous water movement apparatus. Moreover, two different mutations in SLC4A11 (R125H and N639A) inhibited water transport, while leaving plasma membrane expression intact. Finally, treatment with stilbene disulfonate compounds inhibited water movement in SLC4A11-expressing cells, with a half maximal concentration in line with the previously established binding affinity of stilbene disulfonates for SLC4A11 (29). This strongly supports a direct effect of stilbene disulfonates on SLC4A11-mediated water movement.

The only water channel proteins reported thus far are members of the MIP family, which includes mammalian AQP proteins. Some water can, however, move along with solute molecules transported across the plasma membrane (33,34). Indeed, carbohydrate transporters are thought to accumulate water as a consequence of the osmotic gradient developed as they transport solute (34,35). In the case of the Na^+^/glucose co-transporter, SGLT1, the water-flux amounts to 200–260 water molecules for each sugar transported (36), which corresponds to 10^4^ H_2_0.s^−1^ for each SGLT1 expressed in *X. laevis* oocytes (37). Furthermore, water-flux through SLC4A11 is 10^3^-fold faster than water movement reported for SGLT1. Also we saw no water-flux associated with hCNT3, indicating that water-flux is not a common feature of membrane transport proteins. SLC4A11 water movement is thus clearly distinct from that reported for some solute transporters and more closely resembles water movement facilitated by MIP proteins.

Two mechanisms can be invoked to explain the increased rate of cell swelling found in SLC4A11-expressing cells: (i) increased water-flux through the protein and (ii) increased influx of solute into the cell, coupled to parallel water-flux. Swelling assays in HEK293 cells were performed in medium containing (in mm) the solutes 90 NaCl, 5.4 KCl, 0.4 MgSO_4_, 2 CaCl_2_, 10 HEPES, 5.5 glucose and varied mannitol. Mannitol is membrane-impermeant. The rate of cell swelling was unchanged by the absence of Na^+^, indicating that Na^+^ uptake could not explain the rate of cell swelling. The gradient for K^+^ is outward-directed, so it could not be passively transported inward. In contrast, there are inward-directed gradients for Cl^−^, SO_4_^2−^, Ca^2+^, Mg^2+^, HEPES and glucose. Glucose and SO_4_^2−^ are not present in the ND96 medium used in oocyte swelling assays, ruling them out as required for cell swelling. HEPES is a large (molecular weight 238 Da) sulfonic acid that is unlikely to be transported. We cannot absolutely rule out the possibility that SLC4A11 facilitates Cl^−^, Ca^2+^ or Mg^2+^ flux in order to increase the rate of osmotically induced cell swelling.

We consider this possibility unlikely for several reasons. Osmotically driven cell swelling was observed in oocyte swelling assays in which ND96 medium was used at 20% normal dilution, with osmolarity maintained by mannitol. Under these conditions, the concentrations of Cl^−^, Ca^2+^ and Mg^2+^ are 20, 0.4 and 0.2 mm, respectively. This provides little, if any, electrochemical driving force to accumulate these solutes. Hypo-osmotically induced solute accumulation (mechanism 2) would be dangerous for any cell, as it represents a feed-forward process. In hypo-osmotic medium, the adaptive response for the cell is to release solute, to reduce the driving force for water accumulation. Activation of solute influx in response to hypo-osmotic medium, increases cell swelling, risking rupture of the cell's plasma membrane and is maladaptive. Taken together, the data strongly favour direct facilitation of water movement across membranes.

The asparagine residue within the AQP1 and SLC4A11 NPX sequences has different roles. N639 within an NPS sequence in SLC4A11 was required for full water-flux. The two NPS motifs of aquaporins reside in re-entrant loops from the outside and inside, and meet at the centre of the bilayer, where the Asn of the NPS motifs plays a core role in water transport. Deletion of one NPS motif in AQP1 reduced water-flux by 50% (38). That finding requires cautious interpretation as a three residue deletion could have profound effects on protein structure. Supporting this concern, the AQP1 N76S mutant developed a proton conductance, but mediated a WT water-flux (39). Further differentiating the SLC4A11 and AQP1 NPX sequences, the N639PS sequence in SLC4A11 resides in an extracellular loop region on the basis of alignments with AE1 and a proteolytic site tentatively maps adjacent to the NPS sequence (29). The N639S-SLC4A11 mutation impaired water-flux relative to WT-SLC4A11, supporting a direct role of the protein in mediating water-flux. The balance of evidence, however, indicates that the NPS sequences of SLC4A11 and AQP1 do not have a common role in water permeation.

### Role of SLC4A11 in corneal dystrophies

Our data provide an explanation for the role of SLC4A11 in endothelial corneal dystrophies. The role of the corneal endothelial cells is to reabsorb fluid from the stroma, by a complex and incompletely characterized mechanism (2). In mice and humans, we found that SLC4A11 localizes basolaterally in the corneal endothelium, where it is on the opposite surface from apical AQP1. We propose that SLC4A11 forms the basolateral pathway for movement of water from the corneal stroma into the endothelium, while AQP1 moves water out of these cells into the aqueous humour (Fig. 6B). If loss of SLC4A11-mediated water-flux causes corneal dystrophies, then loss of AQP1 might cause the same phenotype as loss of SLC4A11. Indeed, corneas of *aqp1*-null mice have reduced capacity to recover from hypo-osmotic challenge, but *aqp1*-null mice do not display corneal thickening, indicating they are not prone to oedema under basal conditions (40). Also, rare individuals lacking AQP1 expression have no reported corneal abnormalities (41). This may reflect greater functional redundancy in water movement capacity in the human apical endothelial surface than at the basolateral, such that loss of AQP1 leaves other apical AQP proteins able to mediate water-flux, although no other AQP isoform has been reported in the corneal endothelium.

The relative capacity for water movement at apical and basolateral surfaces of the corneal endothelium is an important issue. We found that SLC4A11 carries a lower water-flux per protein molecule than does AQP1. If the water transport capacities of the two cell surfaces were identical (and SLC4A11 and AQP1 were solely responsible for water-flux at their respective cell surface), then the implication is that the abundance of SLC4A11 would need to be much higher than AQP1. Since the two proteins do not share a common epitope, it is not possible to determine the relative abundance of the two proteins, unfortunately. Considering the role of the corneal endothelial cells in water reabsorption from the stroma, an argument could be made that it makes physiological sense to have lower water permeability at the basolateral surface (where SLC4A11 resides) than at the apical (where AQP1 resides). If the endothelial cell reabsorbs more water at its basolateral surface than it can efflux at its apical surface, it will swell with potentially dangerous complications. Endothelial cells would thus function most safely with limited, regulated water-flux at their basolateral surface and excess water-flux capacity at their apical surface. For this reason, loss of basolateral SLC4A11 function would have a more dramatic phenotype than loss of apical water-flux capacity.

In an independently generated strain of *slc4a11*-null mice, the corneal stroma displays increased levels of sodium relative to *WT* (12). This is consistent with a role of SLC4A11 in both Na^+^ and water movement. Nonetheless, our experiments reveal water-flux driven only by an osmotic gradient, established by varying mannitol concentration.

Beyond the cornea, transmembrane water movement through SLC4A11 may be broadly important in human physiology. In human tissues, SLC4A11 expression is elevated in kidney, salivary glands, thyroid gland and testis (3). In rodents, detailed analysis of SLC4A11 mRNA and protein expression indicated measurable abundance in anterior and posterior corneal epithelia, kidney, stomach, duodenum, pancreas, brain and choroid plexus epithelium (42). Adding further complexity, human SLC4A11 occurs as three alternate transcripts, encoding for proteins with common C-terminal 860 amino acids, but unique N-terminal sequences of 58, 31 and 15 amino acids for transcripts 1–3, respectively. Differential abundance of these variants across tissues has not been assessed, yet differing function of these variants may explain differential disease presentation across tissues.

The urinary concentrating defect found in *slc4a11^−/−^* mice (12) can be explained by a role of basolateral SLC4A11 in water reabsorption in the kidney's thick ascending limb, where the protein localizes (12). Hearing defects found in individuals with FECD (13) and Harboyan syndrome (6) are mirrored in *slc4a11^−/−^* mice (12,14). These mice manifest normal levels of K^+^ in the inner ear's endolymph and evidence of extracellular oedema in the fibrocytes underlying the stria vascularis (12). While these defects were previously ascribed to presumed defects in solute transport (12), the finding here that SLC4A11 moves water across membrane in response to osmotic gradients also explains the defects in kidney and inner ear.

Yeast and plant orthologues of SLC4A11 are established as borate transport proteins (15,16) and human SLC4A11 has been identified as a borate transporter in one report (4). The biochemical role for borate is in stabilization of cell walls through cross-linking of vicinal diols (17,18). Lacking a cell wall, there is no established biochemical role for borate in mammalian cells. If borate transport has a physiological role in the cornea, it would more likely be in the prevention of toxicity, although borate is reported to have low toxicity in humans (43). That is, cornea accumulates water as a result of high dissolved solute levels in the stroma (1,2). Perhaps this induces accumulation of borate, which would need to be reabsorbed to prevent borate toxicity. If that is the case, SLC4A11 function would be similar to the plant protein, NIP5;1, which is a dual-activity water and borate transporter. Thus far we have been unable, however, to replicate the observation of borate transport by human SLC4A11, although we can observe borate flux through a plant borate transporter (Vilas *et al*., in preparation).

Since the identification of AQP1 (21), transmembrane water transport has been accepted to occur exclusively via related proteins of the MIP family. Here we have found that SLC4A11, which is not phylogenetically related MIP proteins, also facilitates water movement at a rate similar to AQP proteins. We conclude that the corneal fluid accumulation found in genetic diseases of *SLC4A11* arises at least in part from defective water movement by SLC4A11. Therapeutic strategies for these corneal blinding diseases will need to compensate for this lost capacity. SLC4A11 (44) and its orthologues are widely expressed across human tissues and among species, including yeast, plants, birds and fish. The finding that SLC4A11 facilitates transmembrane water-flux calls for a reconsideration of explanations for biological water movement that had previously been attributed only to MIP family proteins.

## MATERIALS AND METHODS

### Protein expression

#### Oocytes

Plasmid DNA was linearized with *NHE*I in the poly-linker region and used to transcribe RNA *in vitro* with T7 RNA polymerase message machine kit (Ambion, Austin, TX, USA), in accordance with the manufacturer's instructions. Twenty-four hours after collagenase digestion, oocytes were re-selected and injected with a nanolitre injector (Drummond Scientific, Broomall, PA, USA), using 0.5 ng of AQP1 mRNA, 4 ng of SLC4A11, 25 ng NIP5;1 and 25 ng hCNT3 mRNAs. Oocytes were returned to ND96 solution (96 mm NaCl, 2 mm KCl, 1.8 mm CaCl_2_, 2.5 mm NaHCO_3_, 2.5 mm NaPyruvate, 1 mm MgCl_2_, 5 mm HEPES, Gentamycin 0.05 g/l Penicillin 0.1 g/l, pH 7.4) and incubated at 18°C for 3–4 days before the experiments.

#### Mammalian cells

Proteins were expressed by transient transfection of HEK293 cells using the calcium phosphate transfection method (45). Cells were grown and transfected in DMEM, supplemented with 5% (v/v) foetal bovine serum, 5% (v/v) calf serum, 1× penicillin–streptomycin–glutamine and kept in an incubator at 37°C and 5% CO_2_ saturation. All experiments involving transfected cells were carried out 40–48 h post-transfection. In all experiments, splicing variant 2 of human SLC4A11 cDNA, encoding an 891 amino acid protein, was used.

### Measurements of water-flux

Oocytes were pre-incubated with 20% ND96 solution adjusted to 220 mOsmol/kg with mannitol for 10 min at room temperature, oocytes were perfused with hypotonic (44 mOsmol/kg) 20% ND96 buffer and the time course of osmotic volume increase was monitored by video microscopy, with images collected every 15 s. Solution osmolarity was assessed with an Advanced Instruments 3D3 osmometer. Image pro plus software (Media Cybernetics, Silverspring, MD, USA) was used to measure the mean diameter of each oocyte image and then its volume. The relative volume increase responses were plotted as a function of time of exposure to hypotonic buffer were fit with a model-independent second order polynomial, and the initial rates of swelling [(d(*V*/*V*_0_)/d*t*] were calculated from the linear component of the fit. Osmotic water permeability (P_f_) values for each oocyte were calculated as reported previously (23) and are presented as mean ± SEM.

HEK293 cells were grown on poly-l-lysine-coated 25 mm glass coverslips and co-transfected with eGFP and HA-SLC4A11, AQP1, hCNT3, R125H SLC4A11, N639A SLC4A11 or pcDNA 3.1 (empty vector) in a 1:8 molar ratio. Coverslips were mounted in a 35 mm diameter Attofluor Cell Chamber (Molecular Probes). During experiments, the chamber was perfused at 3.5 ml/min with isotonic MBSS buffer (90 mm NaCl, 5.4 mm KCl, 0.4 mm MgCl_2_, 0.4 mm MgSO_4_, 3.3 mm NaHCO_3_, 2 mm CaCl_2_, 10 mm HEPES, 5.5 mm glucose, 100 mm d-mannitol, pH 7.4, 300 mOsm/kg) and then with hypotonic (200 mOsm/kg) MBSS buffer, pH 7.4 (same composition as previous but lacking d-mannitol). The chamber was placed on the stage of a Wave FX Spinning Disc Confocal Microscope (Quorum Technologies, Guelph, Canada), with a Yokogawa CSU10 scanning head. The microscope has a motorized XY stage with Piezo Focus Drive (ASI, MS-4000 XYZ Automated Stage) and a live cell environment chamber (Chamlide, Korea), set to 24°C during the duration of the experiment. Acquisition was performed with a Hamamatsu C9100–13 Digital Camera (EM-CCD) and a 20× objective during excitation with the FiTC/eGFP laser (491 nm). eGFP fluorescence data were acquired at 4 points/s. Lasers are from Spectral Applied Research (Richmond Hill, Ontario, Canada). Quantitative image analysis was performed with Volocity 4.2 software (PerkinElmer, Ontario, Canada).

## SUPPLEMENTARY MATERIAL

Supplementary Material is available at *HMG* online.

*Conflict of Interest statement*. None declared.

## FUNDING

Operating support provided by Canadian Institutes of Health Research. J.R.C. is a Scientist of the Alberta Heritage Foundation for Medical Research (AHFMR) and G.V. was supported by a postdoctoral fellowship of AHFMR. J.D.Y. is an AHFMR Senior Investigator. S.L. was supported by a graduate studentship from the International Research Training Group in Membrane Biology. Funding to pay the Open Access publication charges for this article was provided by Canadian Institutes of Health Research.

## Supplementary Material

Supplementary Data
