# Supplementary material for: Transmembrane water-flux through SLC4A11: a route defective in genetic corneal diseases
Source: Hum Mol Genet. 2013 Jun 27;22(22):4579–90. doi: 10.1093/hmg/ddt307 (PMC3889808; doi:10.1093/hmg/ddt307)
Supplement: Supplementary Data [file supp_22_22_4579__index.html]

Transmembrane water-flux through SLC4A11: a route defective in genetic corneal diseases — Transmembrane water-flux through SLC4A11: a route defective in genetic corneal diseases — Supplementary Data 

# Transmembrane water-flux through SLC4A11: a route defective in genetic corneal diseases

## 

Supplementary Data

**Files in this Data Supplement:**

- Supplementary Data - Doc file
