# Supplementary material for: Transmembrane water-flux through SLC4A11: a route defective in genetic corneal diseases
Source: Hum Mol Genet. 2013 Jun 27;22(22):4579–90. doi: 10.1093/hmg/ddt307 (PMC3889808; doi:10.1093/hmg/ddt307)

**SUPPLEMENTARY MATERIALS AND METHODS**

**cDNA cloning**

The cloning of the N-terminally hemagglutinin (HA) tagged SLC4A11 into pcDNA 3.1 mammalian expression vector was reported earlier. *Arabidopsis* NIP5;1 was provided by the Arabidopsis biological resource centre (The Ohio State University, Columbus, OH). Human AQP1 N-terminally tagged with RFP and human CNT3 were provided by Dr. Todd Alexander and Dr. James Young, respectively . Using standard methods, we cloned the indicated cDNAs into the pGEMHE vector, which provides the necessary 5' and 3' untranslated regions (UTR) from *Xenopus* α-globin or the mammalian expression vector pcDNA3.1.

**Preparation of oocyte expression constructs**

The DNA sequence corresponding to the hemagglutinin (HA) tag, amino acids YPYDVPDYA, was added to the 5’ end of NIP5;1, SLC4A11 and AQP1 by PCR-based sub-cloning and is underlined in the primers. All cDNAs were cloned into the pGEMHE *X. laevis* expression vector. For NIP5;1, the following primers were used: 5’-GC GGTACCATGTACCCCTACGACGTGCCCGACTACGCCGGGGCTCCACCGGCTGAG and 5’-CGC GACGTGTGCCCAAGGGAAGTGCCT-3’ containing the cloning restriction sites *KpnI* and *BtgI*, respectively. HA tag sequence was added to SLC4A11 cDNA using the following primers: 5’-TCCCCCCGGGGCCACCATGTACCCTTACGACGTACCTGACTACGCAGGAAGCCAGGTCGGGGG-3’ and 5’-GCTCTAGACAGGCTGAGGTTGAAAAAGG-3’ and then sub-cloning this fragment into SLC4A11 sequence using the restriction enzymes *XmaI* and *BbvCI*. HA sequence was introduced in AQP1 using the primers: 5’-GCCCCGGGATGTACCCCTACGACGTGCCCGACTACGCCGGGTACGCCGCCAGCGAGTTCAAGAAG-3’ and 5’-CGCCCGCGGAGCCAAAGGACCGAGCAG-3’ and the fragment sub-cloned using *XmaI* and *BtgI*.

**Preparation of mammalian expression constructs**

An untagged version of human AQP1 was generated by PCR and cloned into the pcDNA 3.1 mammalian expression vector using the following primers: 5’-CGGGATCCGACCATGGCCAGCGAGTTCAAGAAGA-3’ and CCCTCGAGCTATTTGGGCTTCATCTCCAC-3’ containing the cloning restriction sites *BamHI* and *XhoI*, respectively. hCNT3 in the mammalian expression construct was generated by excision of hCNT3 from yeast expression vector pYPGE15 with *KpnI* and *EcoRI*, and ligation into pcDNA3.1.

**Preparation of A639PS SLC4A11 mutant**

A missense mutation of SLC4A11, where Asp^639^ was replaced by Ala, was generated using the PCR-based Quikchange Lightning Site-Directed Mutagenesis Kit (Stratagene, La Jolla, CA) and HA-tagged SLC4A11 cDNA as template, following manufacturer’s protocol. The mutation (underlined) was introduced in the HA-SLC4A11 sequence using the following primers: 5’- GCAAGTTCCGCTACGCCCCCAGCGAGAGCC-3’ (sense) and 5’- GGCTCTCGCTGGGGGCGTAGCGGAACTTGC-3’ (anti-sense). After the amplification step the original, unaltered cDNA template, was digested with DpnI restriction enzyme. The identity and fidelity of all the clones were confirmed by sequencing.

***Xenopus laevis* oocyte isolation and maintenance**

Adult female *Xenopus laevis* were obtained from Xenopus One (Ann Arbor, MI), housed in an established frog colony, and fed regular frog brittle twice wkly. For the removal of oocytes, frogs were anaesthetized by immersion in 0.5% (w/v) MS222 (Tricaine methane-sulfonate/Ethyl 3-aminobenzoate methanesulfonate salt, Sigma-Aldrich) until unresponsive to a painful stimulus. A 1 cm incision was made in the abdominal wall through both the skin and muscle layers, and a lobe of ovary containing oocytes was excised. The wound was closed in two layers and the animal was allowed to recover from anaesthesia. Ovarian follicles were removed and digested by gentle agitation in ND96 solution (96 mM NaCl, 2 mM KCl, 1.8 mM CaCl_2_, 1 mM MgCl_2_, 10 mM Hepes, pH 7.4), containing 2 mg/ml collagenase type 1 (Worthington, Lakewood, NJ) twice for approximately 60 min. After digestion, healthy oocytes at stage VI were manually selected based upon size and uniformity of color and incubated at 18 °C in ND96 solution supplemented with 0.1 mg/ml penicillin and 0.05 mg/ml gentamicin sulfate. All animal procedures conformed with, and performed under an approved University of Alberta protocol (#599/06/10/C).

**Preparation of Oocyte Total Membrane Fractions**

Oocytes were rinsed and disrupted in ND96 supplemented with protease inhibitor cocktail by pipetting up and down 20 times. Homogenates were centrifuged at 250 x g for 10 min at 4 °C to pellet cell debris, and the resulting supernatant was centrifuged at 16,000 x g for 20 min at 4 °C to produce a membrane-enriched fraction. Pellets were resuspended in ND96 buffer (2 µl solution/oocyte), containing protease inhibitor cocktail and frozen until use.

**Immunoblots**

Immunoblots were performed using enriched membrane fractions isolated from *X. laevis* oocytes or detergent-solubilized HEK 293 extracts. Equal amounts of total protein were separated by SDS-PAGE and electrotransferred onto Immobilon-P PVDF membranes (Millipore Corporation, MA, USA) for 1 h at a constant current of 400 mA. After transfer, membranes were rinsed in TBS (0.15 M NaCl, 50 mM Tris-HCl, pH 7.5) and incubated with TBS-TM (TBS containing 0.1% (v/v) Tween-20 and 5% (w/v) skim milk) for 1 h at room temperature with gentle rocking to block nonspecific binding. Membranes were then incubated for 16 h at 4 °C with gentle rocking in the presence of either mouse anti-HA, rabbit anti-AQP1, rabbit anti-hCNT3 or rabbit anti-Caveolin at 1:1500, 1:2000, 1:1000 and 1:2500 dilution in TBS-TM, respectively. After successive washes with TBS and TBS-T (TBS containing 0.1% (v/v) Tween-20), the membranes were incubated with a 1:5000 dilution of the appropriate HRP-conjugated secondary antibodies in TBS-TM for 1 h at 20 °C and further washed with TBS and TBS-T. Proteins were detected using Western Lightning™ Chemiluminescence Reagent Plus (PerkinElmer Las, Inc., MA, USA) and visualized using a Kodak Image Station 440CF (Kodak, NY, USA). Quantitative densitometric analyses were performed using Kodak Molecular Imaging Software v4.0.3 (Kodak, NY, USA).

**Cell surface biotinylation assays**

HEK293 cells were transiently transfected with were transfected with cDNA encoding AQP1, SLC4A11 and hCNT3. Samples were processed to determine the efficiency of cell surface processing, as described previously .

**Immunohistochemistry of cornea**

Human cornea samples were obtained from surgical remnants during corneal transplantation. Human and mouse cornea sections were deparaffinized with two 5 min washes in CitriSolv clearing agent (Fisher Scientific, ON, Canada). Sections were rehydrated by successive washes with decreasing ethanol (EtOH) concentrations (two washes of 5 min each with 100% EtOH, 1 wash of 2 min with 70% EtOH, 1 wash of 2 min with 50% EtOH) and a final 2 min wash with deionized water. The slides were then placed in 50 ml Falcon tubes containing pre-heated 0.05% Tween-20 in Tris-EDTA, pH 9.0 and placed in boiling water for 20 min. After cooling for 10 min under running water, the slides were removed from the Falcon tubes, washed twice, for 5 min, in TBS-TI (0.025% Triton X-100, 0.15 M NaCl, 50 mM Tris, pH 7.5) and blocked for 2 h at 22°C in TBS (0.15 M NaCl, 50 mM Tris, pH 7.5), containing 10% non-immune rabbit serum and 1% BSA. Human cornea sections were incubated 16 h, at 4°C, with 1:100 dilutions of rabbit anti-human SLC4A11 (described below) or rabbit anti-AQP1 (Santa Cruz Biotechnology #20810) or non-immune rabbit serum in TBS containing 1% BSA (TBS-B). In co-immunofluorescence experiments with mouse cornea sections, sections were incubated with 1:100 dilutions of rabbit anti-mouse SLC4A11 (described below) and either goat anti-NHE1 (Santa Cruz Biotechnology # 16097), goat anti-AQP1 (Santa Cruz Biotechnology # 34008), or goat anti-Na^+/^K^+^-ATPase Beta-1 (Santa Cruz Biotechnology #16053). Corneal sections were then subjected to three 5 min washes with TBS-TI. Human corneal sections then received 1 h incubation with 1:1000 dilution of Alexa 594-conjugated chicken anti-rabbit antibody (Molecular probes, Eugene, OR) in TBS-B. For mouse cornea co-immunofluorescence sections were co-incubated for 1 h with 1:1000 dilution each of Alexa 488-conjugated chicken anti-rabbit antibody and Alexa 594-conjugated chicken anti-goat antibody. Cornea sections were washed in the dark for 15 min with TBS to remove excess antibody, and mounted in Prolong Gold Anti-fade Solution (Invitrogen, Burlington, ON, Canada), containing the DNA-specific fluorescent dye 4’,6-diamidino-2-phenylindole (DAPI). Confocal images were generated using an IX81 motorized inverted microscope (Olympus, Burlington ON) with a MS-2000 motorized XY stage harbouring a piezo Z insert that has 100 µm travel (Advanced Scientific Instrumentation, Eugene OR, USA) and a CSU 10 spinning disk confocal scan-head (Yokogawa, Tokyo, Japan). Samples were observed through 10X/0.3 Ph1, 20X/0.75 Dry and 60X/1.42 PlanApo oil immersion objectives. Image capture was performed with a C9100-13 EM-CCD Digital Camera (Hamamatsu, Hamamatsu City Japan), using Volocity software (Perkin Elmer, Mississauga ON).

**Whole Mount Oocyte Immunohistochemistry**

Three days post-mRNA injection, intact oocytes were fixed in 4% paraformaldehyde for 15 min at 4 °C, followed by three washes with PBS containing 50 mM NH_4_Cl. Oocytes were permeabilized for 1 h in 0.1% Triton X-100 and washed with PBS. Samples were then blocked 30 min with 2% bovine serum albumin (BSA) in PBS, followed by overnight incubation with a mouse monoclonal anti-HA antibody (clone 16B12, Covance, Emeryville, CA) diluted in blocking buffer. Oocytes were washed in PBS for 30 min, followed by incubation for 1 h with a chicken anti-mouse Alexa 488 secondary antibody (Molecular probes, Eugene, OR) diluted in blocking buffer. Oocytes were rinsed in PBS for 30 min and imaged with a 10× objective on a spinning disk confocal microscope (Quorum Technologies inc., Guelph, Ontario), using FiTC/GFP laser (491 nm). The acquisition was performed with a Hamamatsu C9100-13 Digital Camera (EM-CCD) (Bridgewater, NJ) and analyzed with Volocity 4.2 software.

**Generation of *Slc4a11^-/-^* Mice**

Heterozygous *Slc4a11* knockout C57BL/6 mice with a targeted deletion of exons 9-13 of the *Slc4a11* gene were generated under contract by Ozgene (Bentley, Australia). Briefly, mice deficient for *Slc4a11* were generated using the Cre-Lox system , whereby the gene locus was modified by lox P elements flanking exons 9-13 of the *Slc4a11* gene (Figure S8). Upon Cre-mediated deletion a frameshift is introduced, with a premature stop introduced in exon 16. The altered mRNA transcript, lacking the sequences of the trans-membrane helices of the wild type (WT) protein, is likely to be a target for degradation by nonsense mediated decay. Initially heterozygous *Slc4a11* (WT/flox) were generated and then crossed with strain OzCre, which are homozygous for a *Cre* transgene in the ROSA26 locus. Mice resulting from this mating were screened for the presence of *Cre* gene and the deletion of *Slc4a11* exons 9-13. These mice (wt/KO-wt/Cre) were backcrossed to C57BL/6 (wild-type) to eliminate the *Cre* gene and the resulting heterozygous mice were bred to homozygosity and used for genetic and phenotypic analysis. All animal experiments were performed according to the care of experimental animal guideline and all protocols were approved by the Institutional Animal Care and Use Committee of SingHealth (IACUC) and the Singapore General Hospital, Singapore. All aspects of the study were in accordance with the Association for Research in Vision and Ophthalmology (ARVO) Statement on the Use of Animals in Ophthalmic and Vision Research.

*Genotyping*. Genomic DNA was isolated from the mice tails using the Centra Puregene Mouse Tail Kit (Qiagen, Hilden, Germany) according to manufactures protocol. To detect the mutant alleles, the following primer pairs were used (product size 386 bp): F2-5’GCCAAGGTATGGAG AACACC3’ and R1-5’GCACAAACGTGATGGAAATG3’. Wild-type alleles were detected with the following primers (product size 353 bp): F1-5’TCTGGACTTCAACGCCTTCT3’ and R1- 5’GCACAAACGTGATGGAAATG3’.

**Phenotypic analysis of *Slc4a11^-/-^* mice cornea**

*Slit lamp examination. Slc4a11*^+/+^ and *Slc4a11*^-/-^ mice were anesthetised by intra peritoneal (i.p.) injection with a combination of ketamine (20 mg/kg body weight, Parnell Laboratories, Alexandria, Australia) and xylazine (2 mg/kg body weight, Troy Laboratories, Smithfield, Australia) and subjected to slit lamp examination using the Micron III Anterior Segment Imaging (Phoenix Research Laboratories, Pleasanton, CA, USA) according to the manufacture’s protocol. Slit lamp and retro illumination photographs were taken of corneas of 24 and 48 week old mice and three mice were studied per genotype.

*Measurement of corneal thickness in Vivo-* Mice were anesthetised as described above and corneal thickness was measured by *in vivo* confocal microscopy (Heidelberg retina tomography HRT3, Heidelberg Engineering GmbH, Germany). Corneal thickness was determined from z-scans images of external to inner corneal surfaces. The images of the central cornea were recorded by z-scans at a rate around 10 µm/s and analyzed with the Heidelberg Eye Explorer version 1.5.1 software (Heidelberg Engineering GmbH, Germany). Measurements were taken from 5 mice for a given genotype. At least three measurements were obtained for each eye of every animal and corneal thickness was measured in mice ranging in age from 2 to 60 weeks.

*Corneal endothelial staining and cell density measurement-* Corneas were excised outside the limbus from enucleated eyes. The corneas were washed in 1x PBS and stained with 1% alizarin red solution (Sigma-Aldrich, St. Louis, MO, USA) for 8 min, washed twice in 1x PBS, and placed under a cover slip for light microscopy (Axioplan2, Zeiss, Stuttgart, Germany). The images were taken from the area corresponding to the central cornea and processed using image analysis software (Axiovisions 4.0, Zeiss, Stuttgart, Germany). The cells were counted, using Adobe Photoshop and the cell density calculated following previously described methods . Mean ± SEM values and statistical significance were calculated using Graphpad Prism Software (La Jolla, California).

*Scanning electron microscopy-* The eye globes were immersed in a fixative solution, containing 2.5% glutaraldehyde (Sigma, St. Louis, MI) in 0.1M sodium cocodylate, pH 7.4 (EMS, Hatfield, PA) overnight at 4 ºC. Following excision from the eye globes, the corneas were washed three times in distilled water for 10 min each. They were then immersed in 1% osmium tetroxide (FMB, Singapore) for 2 h at 22 °C. Following this, corneas were dehydrated in a graded ethanol series of 25%, 50%, 75%, 95% and 100% each for 10 min, with the 100% being performed twice. The samples were then dried in a critical point dryer (BALTEC, Balzers, Liechtenstein) and mounted on SEM stubs using carbon adhesive tabs. Samples were then sputter coated with a 10 nm thick layer of gold (BALTEC) and examined with a JSM-5600 scanning electron microscope (JEOL, Tokyo, Japan) at 15 W.

**Anti-SLC4A11 sera**

Affinity purified rabbit anti-SLC4A11 antibodies, raised against respective antigenic peptide sequences SQNGYFEDSSYYKC and SSLHTHRHPQPPKC, corresponding to of the cytoplasmic N-terminal domain amino acids 37-50 of human SLC4A11 and 292-305 of mouse SLC4A11, were prepared by Genscript (Piscataway, NJ, USA)

**Statistics for data analysis**

Data were analyzed using ANOVA. *P* <0.05 was considered to be statistically significant. Data are presented as the mean ± SEM.

**SUPPLEMENTARY FIGURE LEGENDS:**

**Figure S1.** Phylogeny of SLC4A11. SLC4A11 evolutionary relationship with bicarbonate transporters, MIP and Boron Transporter families. Amino acid sequences of *Arabidopsis thaliana* PIP2.2, TIP2.2, NIP5;1, BOR1, *S. cerevisiae* BOR1 and human AQP1, SLC4a1, 2, 3 and 11 were aligned, using the ClustalW2 algorithm (http://www.ebi.ac.uk/Tools/clustalw2/index.html). The program generated a phylogram showing the relative evolutionary relationships between the proteins.

**Figure S2.** Effect of osmolarity changes on intracellular pH. HEK 293 cells, grown on glass coverslips and transiently transfected with cDNA encoding SLC4A11, AQP1 or hCNT3, were incubated for 15 min with the pH-sensitive dye BCECF-AM. (**A**) The coverslips were then placed in a fluorescence cuvette and perfused alternately with isotonic (black bar) and hypotonic (white bar) medium. Fluorescence changes, associated with variations in intracellular pH (pHi), were monitored in a fluorimeter using excitation wavelengths 440 and 502.5 nm and emission wavelength 528.7 nm. Blue, red and brown traces represent results from SLC4A11, AQP1 and hCNT3, respectively. (**B**) Rates of pH_i_ change during perfusion with hypotonic medium were calculated from experiments similar to that shown in (A) by linear regression of the initial rate of pH_i_ change after switching perfusion to hypotonic buffer. (**C**) Cell lysates were prepared from the remaining cells used in (**A**). Samples were subjected to immunoblotting, and SLC4A11, AQP1 and hCNT3 were detected using mouse monoclonal anti-HA, rabbit anti-AQP1 and rabbit anti-hCNT3 antibodies, respectively. Error bars represent standard error (n = 3).

**Figure S3.** Efficiency of protein cell surface targeting in HEK293 Cells. (**A)** HEK293 cells were transfected with cDNA encoding AQP1, SLC4A11 and hCNT3. Forty-eight h later, cells were incubated with membrane-impermeant Sulpho-NHS-SS-biotin (SNSB) and lysed. One half of the lysate was incubated with streptavidin-agarose resin to capture biotinylated proteins. The other half (Total) and the unbound fraction after removing resin were processed for immunoblotting with the corresponding antibodies, as indicated (pAb, polyclonal antibody; mAb, monoclonal antibody). GAPDH was used as an internal control for the assay. (**B)** Plasma membrane localized fraction of the proteins was calculated and GAPDH represents the background for this assay. Error bars represent standard error (n=3-5). * represents significant difference (P<0.05) between hCNT3 and all other proteins and # represents GAPDH and all other proteins.

**Figure S4.** Expression and cell surface trafficking of N639A SLC4A11. HEK293 cells were transfected with cDNAs encoding *N*-terminally HA-tagged WT, R125H and N639A SLC4A11. (**A**) Forty-eight h later, cells were incubated with membrane-impermeant Sulpho-NHS-SS-biotin (SNSB) and lysed. One half of the lysate was incubated with streptavidin-agarose resin to capture biotinylated proteins. The other half (Total) and the unbound fraction after removing resin were processed for immunoblotting with the corresponding antibodies, as indicated. GAPDH was used as an internal control for the assay. (**B)** Plasma membrane localized fraction of the proteins was calculated and GAPDH represents the background for this assay. Error bars represent standard error (n=3). * represents GAPDH and all other proteins.

# **Figure S5.** Water flux through SLC4A11 is sodium-independent. HEK293 cells were transiently co-transfected with eGFP cDNA and empty vector or SLC4A11 cDNA. Cells were perfused alternately with Na^+^-containing iso-osmotic (black bars) and hypo-osmotic (white bars) buffers in the absence **(A)** or presence **(B)** of 5 µM 5-(N-Ethyl-N-isopropyl)-amiloride (EIPA). **(C)** Cells were alternatively perfused with Na^+^-free iso- and hypo-osmotic buffers (where NaCl and NaHCO_3_ were substituted with choline chloride and KHCO_3_, respectively) in the presence of 5 µM EIPA. **(D)** eGFP fluorescence in regions of interest was measured digitally and the rates of fluorescence change were calculated by linear regression of the corresponding fluorescence values during the first 15 s of perfusion with hypo-osmotic buffer. Rates were corrected for activity of vector-transfected cells. Data represent the mean ± SE of three independent swelling experiments with 30-40 cells measured per assay. N.S. not significant, p>0.05.

**Figure S6.** Targeting strategy to delete exons 9-13 of the *Slc4a11* gene. **(A)** Exons 2-21 of the wild-type allele indicating the location of the 5’ and 3’ homology arms in the gene targeting vector, the location of two probes (5’ probe and 3’ probe) outside the gene targeted region, and the location of one internal probe. Also shown are the restriction sites used for Southern blotting to distinguish wild-type, targeted, and deleted alleles. **(B),** The targeted allele after recombination has inserted lox P sites and the Neo gene cassette. **(C)**, The deleted allele produced by breeding to Cre recombinase-expressing mice, thus deleting exons 9-13 and the Neo gene cassette.

**Figure S7.** Screening for gene targeting at the Slc4a11 locus. **(A)** Exons 2-21 of the wild-type allele (top) and the deleted allele without exons 9-13 (bottom) are shown. Also indicated are the location of the internal probe (en probe) and the PCR primers used for genotyping. The en probe detects a 5.1 kb SpeI fragment specific for the targeted allele (the 3’ homology arm creates an additional SpeI site) or a 22.5 kb fragment specific for the wild-type allele. **(B)** Southern hybridization using the en probe on mouse tail DNA of wild type (*slc4a11*^+/+^) and heterozygous (*slc4a11^+/-^*) mice. **(C)** Genotyping of wild type (*slc4a11*^+/+^), heterozygous (*slc4a11^+/-^*) and KO (*slc4a11*^-/-^) mice by PCR.

**Figure S8.** Scanning electron micrographs of corneal endothelia from wildtype and *slc4a11^-/-^* mice. Sequential SEM of the mouse endothelial cells showing regular pleomorphism and polymegathism in the *slc4a11*^+/+^ mouse from 12 wk to 60 wk. Sequential SEM in the *slc4a11^-/-^*mouse showed increasing swelling of the endothelial cell layer from 32-60 wk. The endothelium showed increasing distortion of the cell borders with an increased amount of polymegathism from 32-60 wk. Magnification, X700. Scale bar, 20 µm.

**Figure S9.**Control immunofluorescence of human cornea sections. Confocal immunofluorescence microscopy images of paraffin-embedded human corneal epithelium (top) and endothelium (bottom). Cornea sections were incubated with anti-SLC4A11 (left panel) or non-immune rabbit serum (right panel), followed by goat anti-rabbit IgG conjugated with Alexa Fluor 594 (red). Nuclei were detected with DAPI (blue). Scale bars represent 10 µm.

**Figure S10.** Immunofluorescence localization of SLC4A11 and AQP1 in *wt* and *slc4a11^-/-^* mouse cornea. Wild type and *slc4a11*^-/-^ mouse corneas were embedded in paraffin and incubated with rabbit anti-mouse SLC4A11 and goat anti-AQP1. Sections were then incubated with chicken anti-rabbit IgG conjugated with Alexa Fluor 488 (green) and chicken anti-goat IgG conjugated with Alexa Fluor 594 (red). Nuclei were detected using DAPI (blue). Scale bar represents 10 µm.

**Figure S11.** Immunofluorescence localization of SLC4A11, NHE1 and Na^+/^K^+^-ATPase localization in *wt* and *slc4a11*^-/-^ mouse cornea. The paraffin embedded mouse corneas from wild type and *slc4a11*^-/-^ mouse were incubated with rabbit anti-mouse SLC4A11, goat anti-NHE1 **(A)** or goat anti-Na^+/^K^+^-ATPase **(B)**. Sections were then incubated with chicken anti-rabbit IgG conjugated with Alexa Fluor 488 (green) and chicken anti-goat IgG conjugated with Alexa Fluor 594 (red). Nuclei were detected using DAPI (blue). Scale bar represents 10 µm.

**REFERENCES**

1 Vithana, E.N., Morgan, P., Sundaresan, P., Ebenezer, N.D., Tan, D.T., Mohamed, M.D., Anand, S., Khine, K.O., Venkataraman, D., Yong, V.H. *et al.* (2006) Mutations in sodium-borate cotransporter SLC4A11 cause recessive congenital hereditary endothelial dystrophy (CHED2). *Nature Genetics*, **38**, 755-757.

2 Zhang, J., Tackaberry, T., Ritzel, M.W., Raborn, T., Barron, G., Baldwin, S.A., Young, J.D. and Cass, C.E. (2006) Cysteine-accessibility analysis of transmembrane domains 11-13 of human concentrative nucleoside transporter 3. *Biochem J*, **394**, 389-398.

3 Vilas, G.L., Loganathan, S., Quon, A., Sundaresan, P., Vithana, E.N. and Casey, J.R. (2012) Oligomerization of SLC4A11 protein and the severity of FECD and CHED2 corneal dystrophies caused by SLC4A11 mutations. *Human Mutation*, **33**, 419-428.

4 Koentgen, F., Suess, G. and Naf, D. (2010) Engineering the mouse genome to model human disease for drug discovery. *Methods Mol Biol*, **602**, 55-77.

5 Jun, A.S., Chakravarti, S., Edelhauser, H.F. and Kimos, M. (2006) Aging changes of mouse corneal endothelium and Descemet's membrane. *Exp Eye Res*, **83**, 890-896.


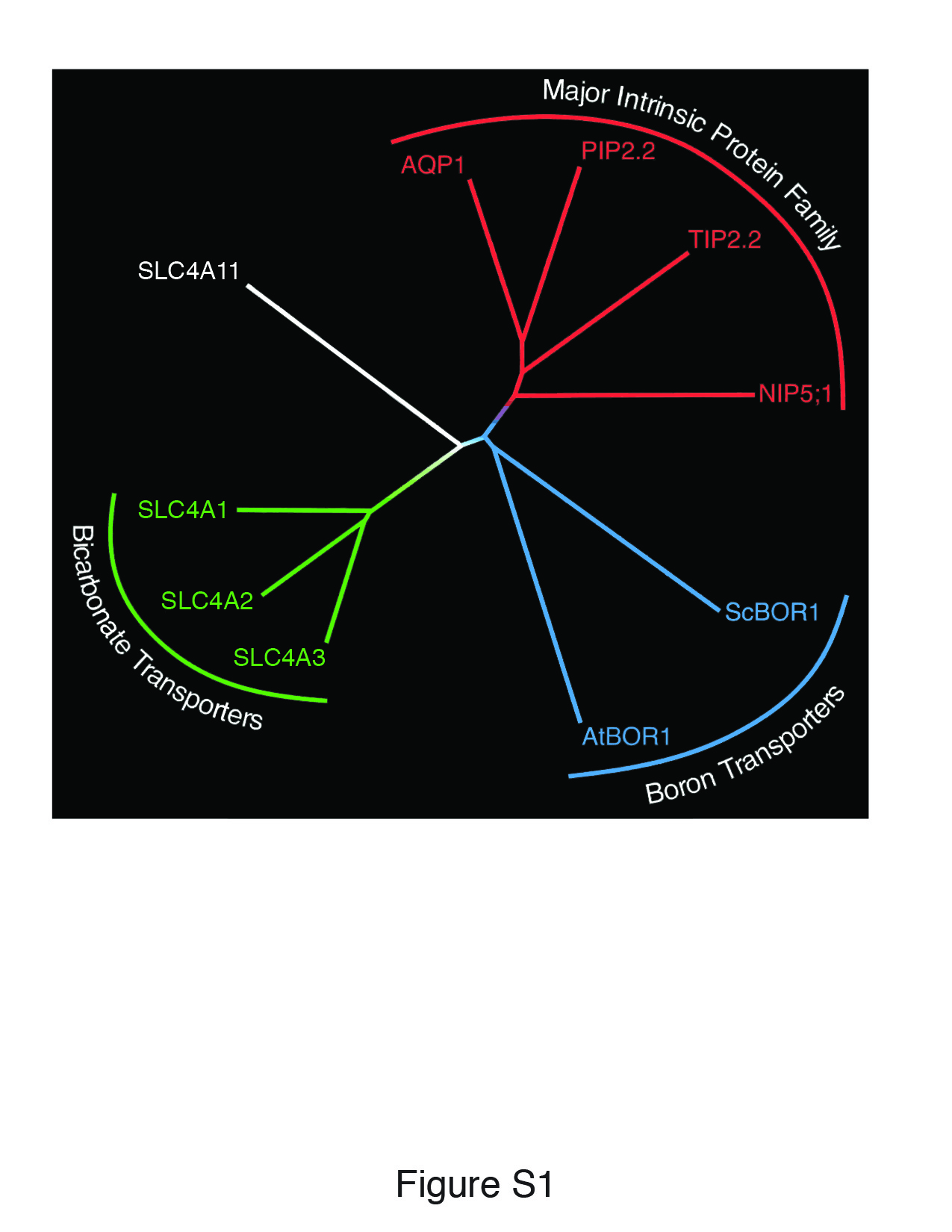


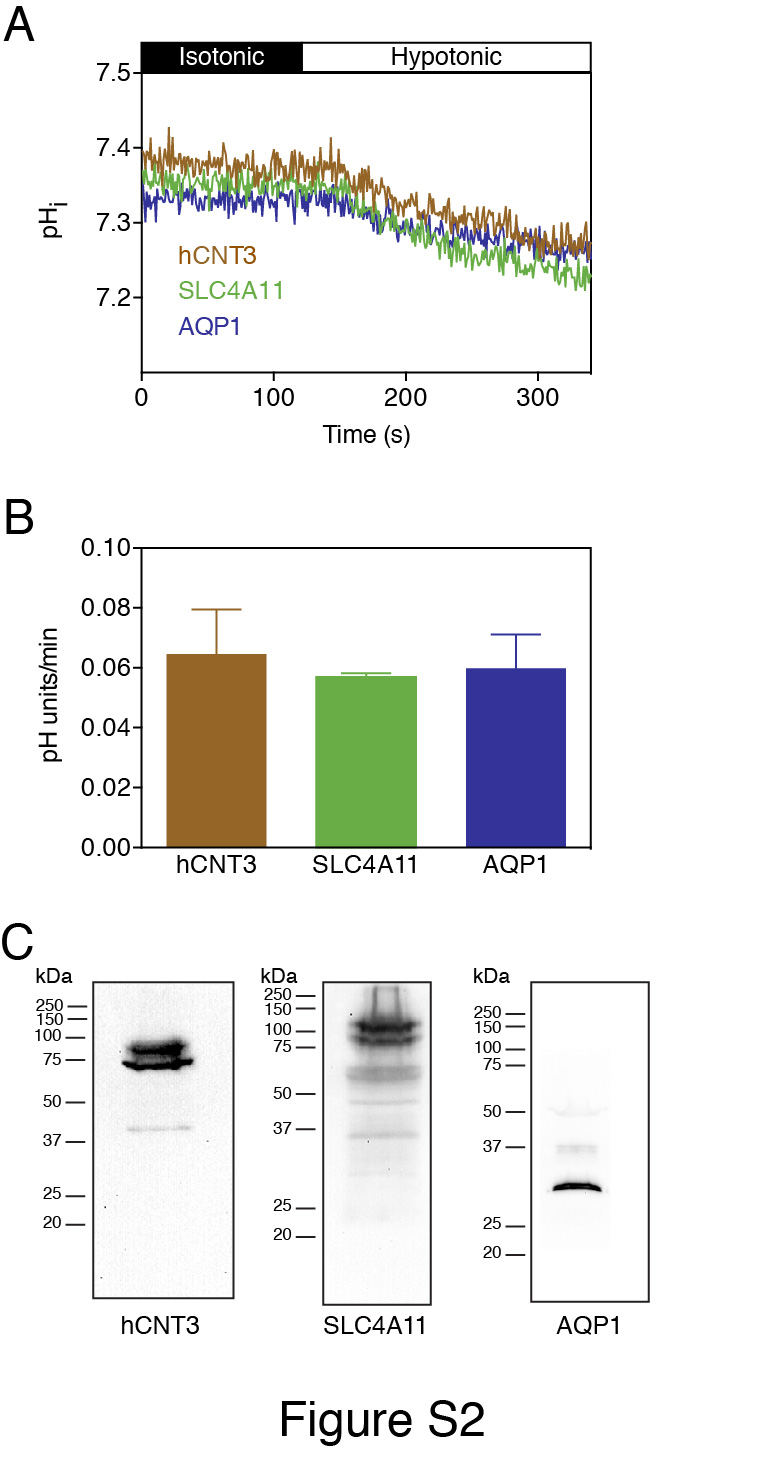


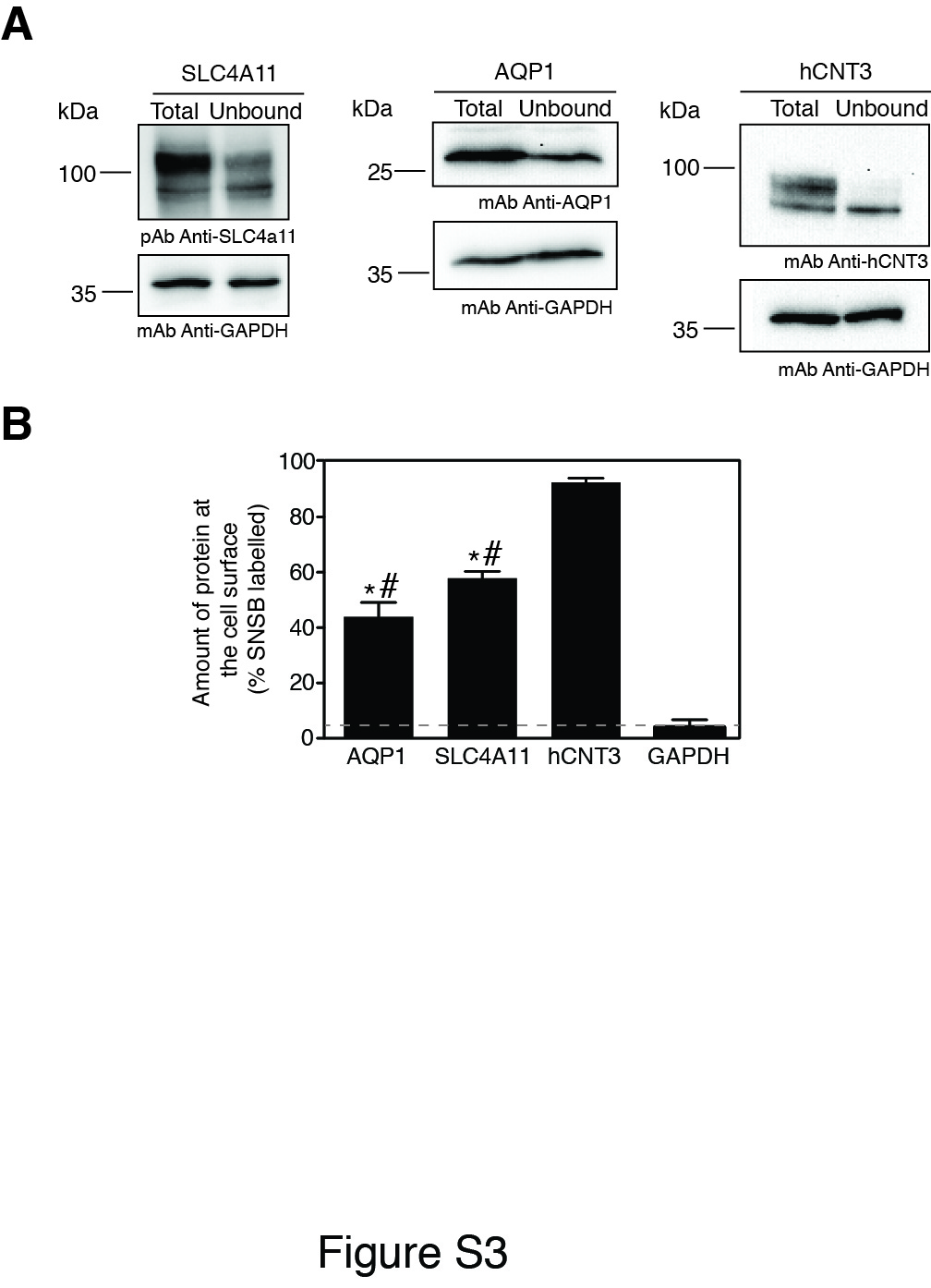


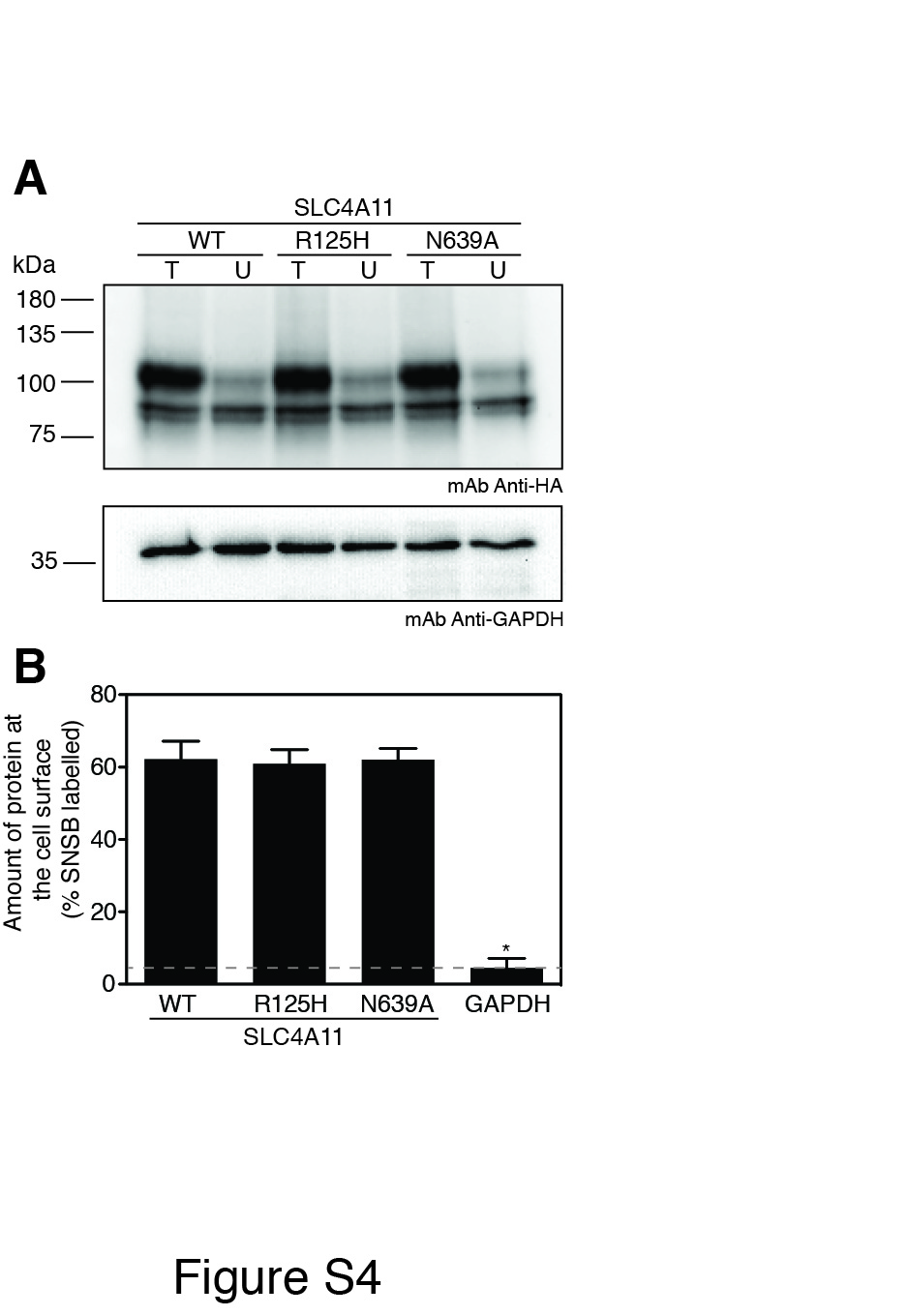


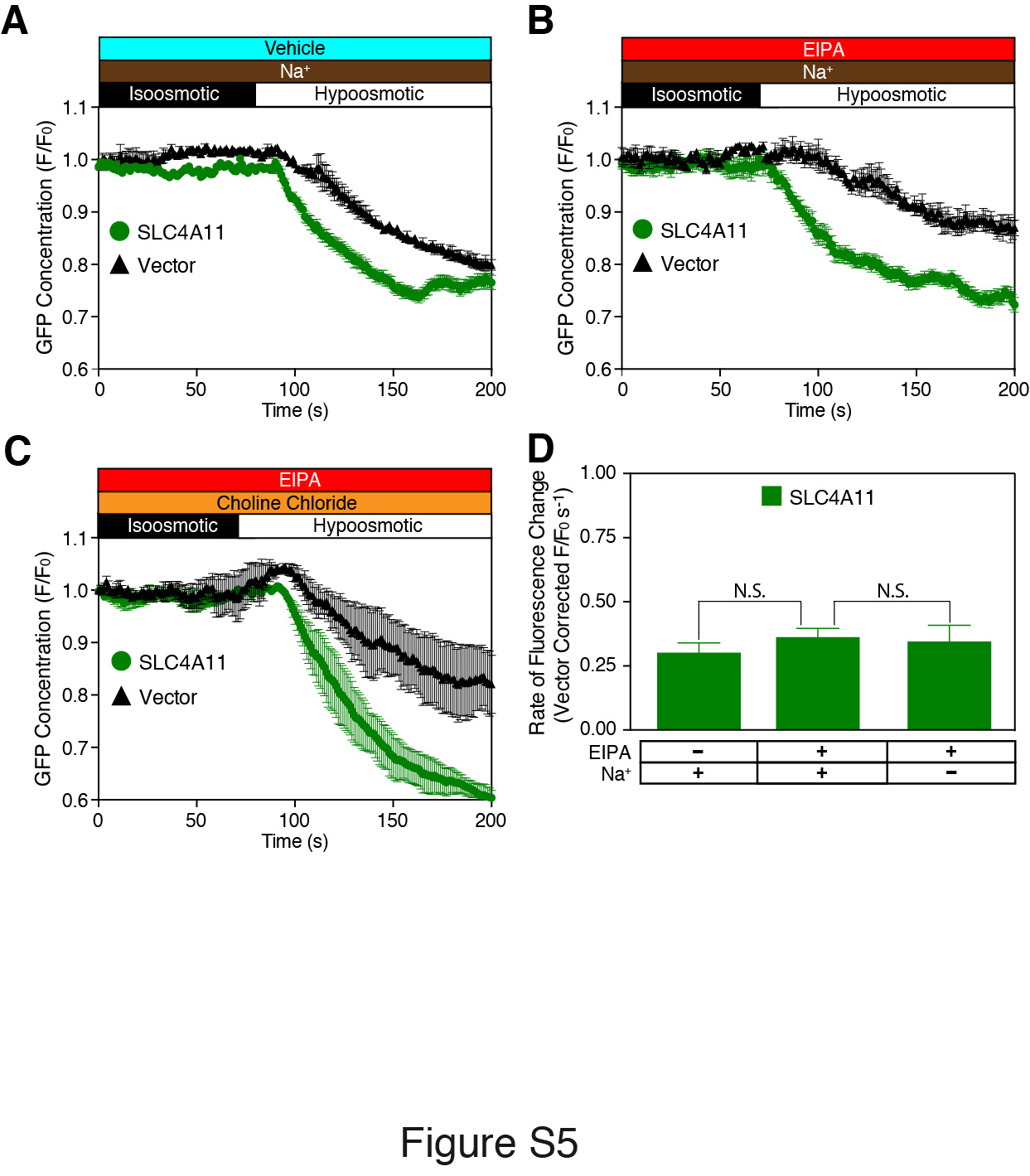


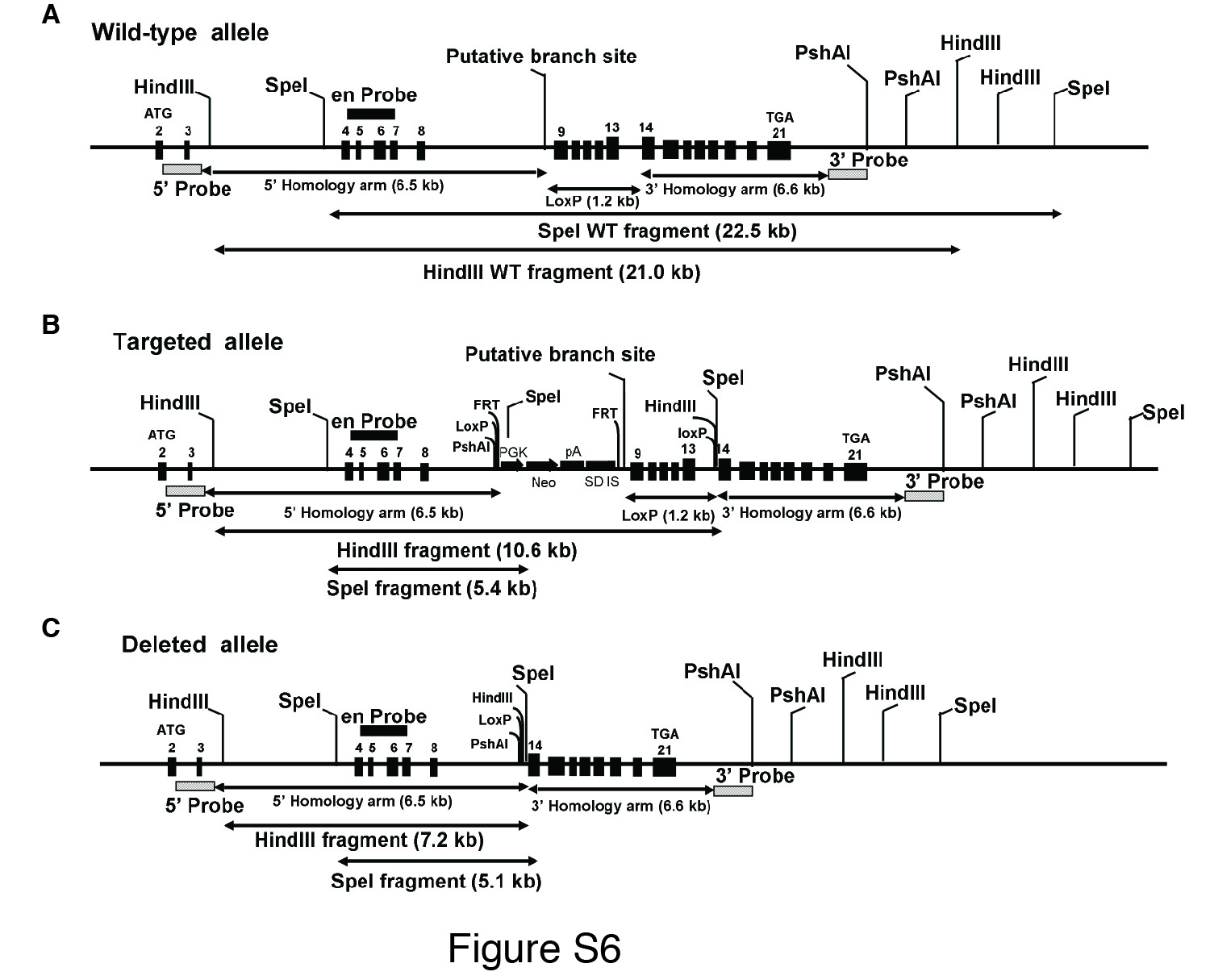


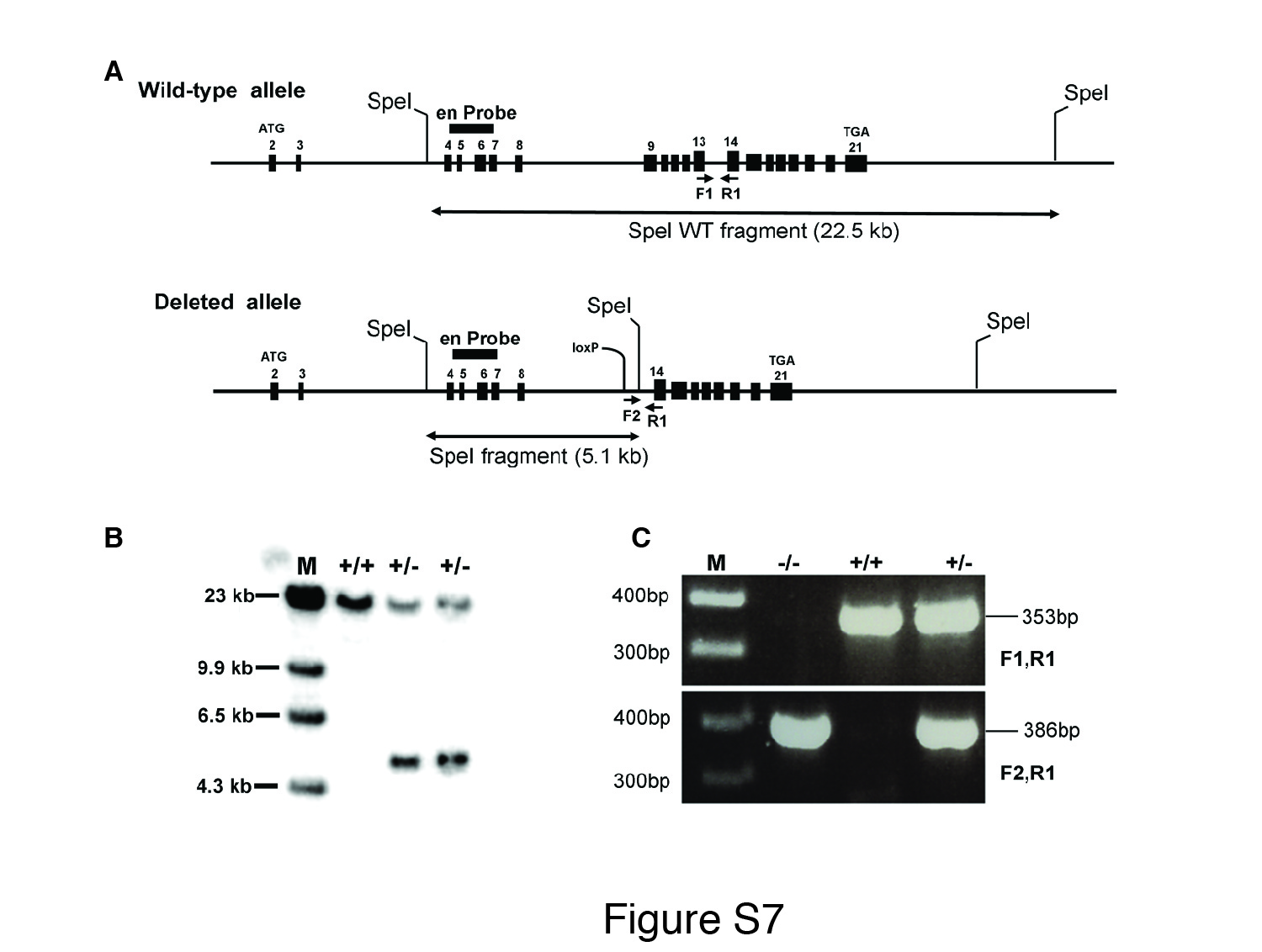


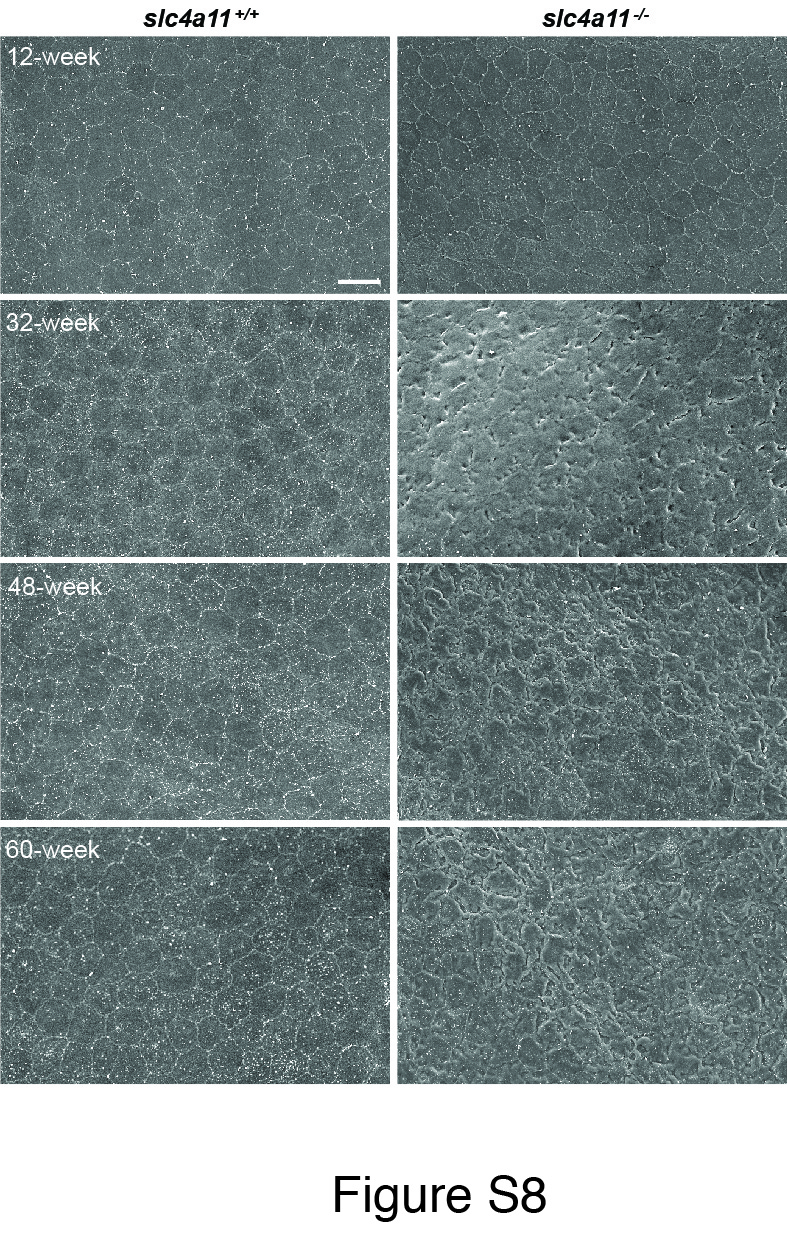


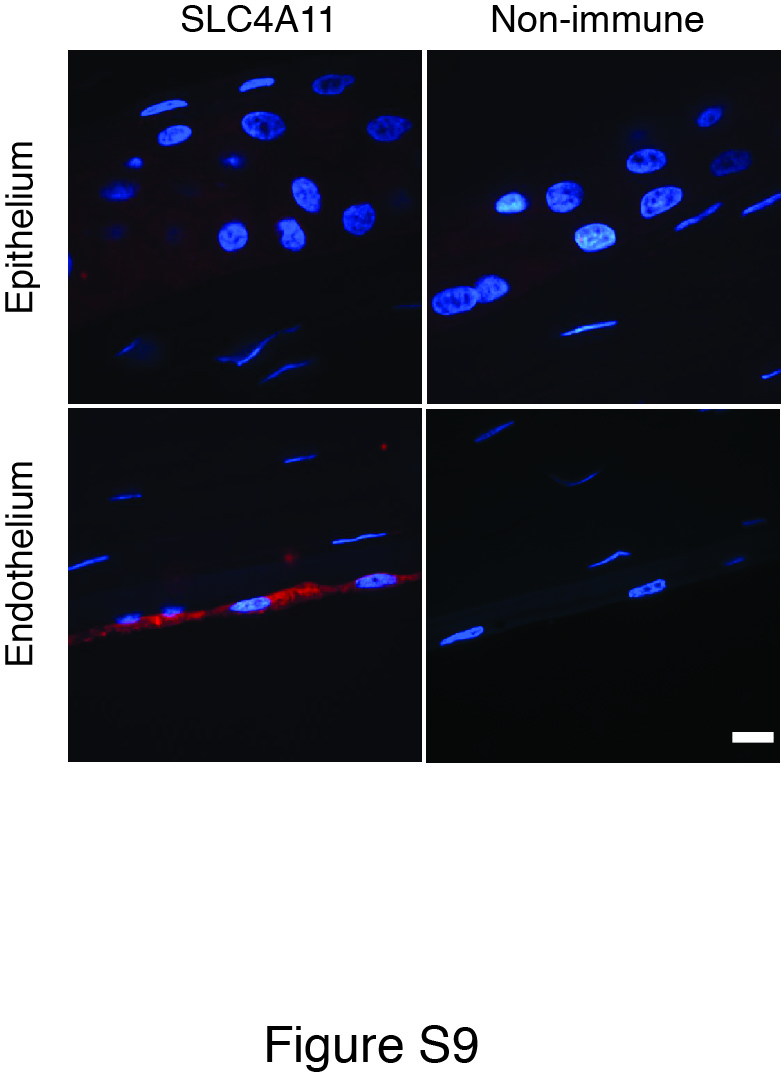


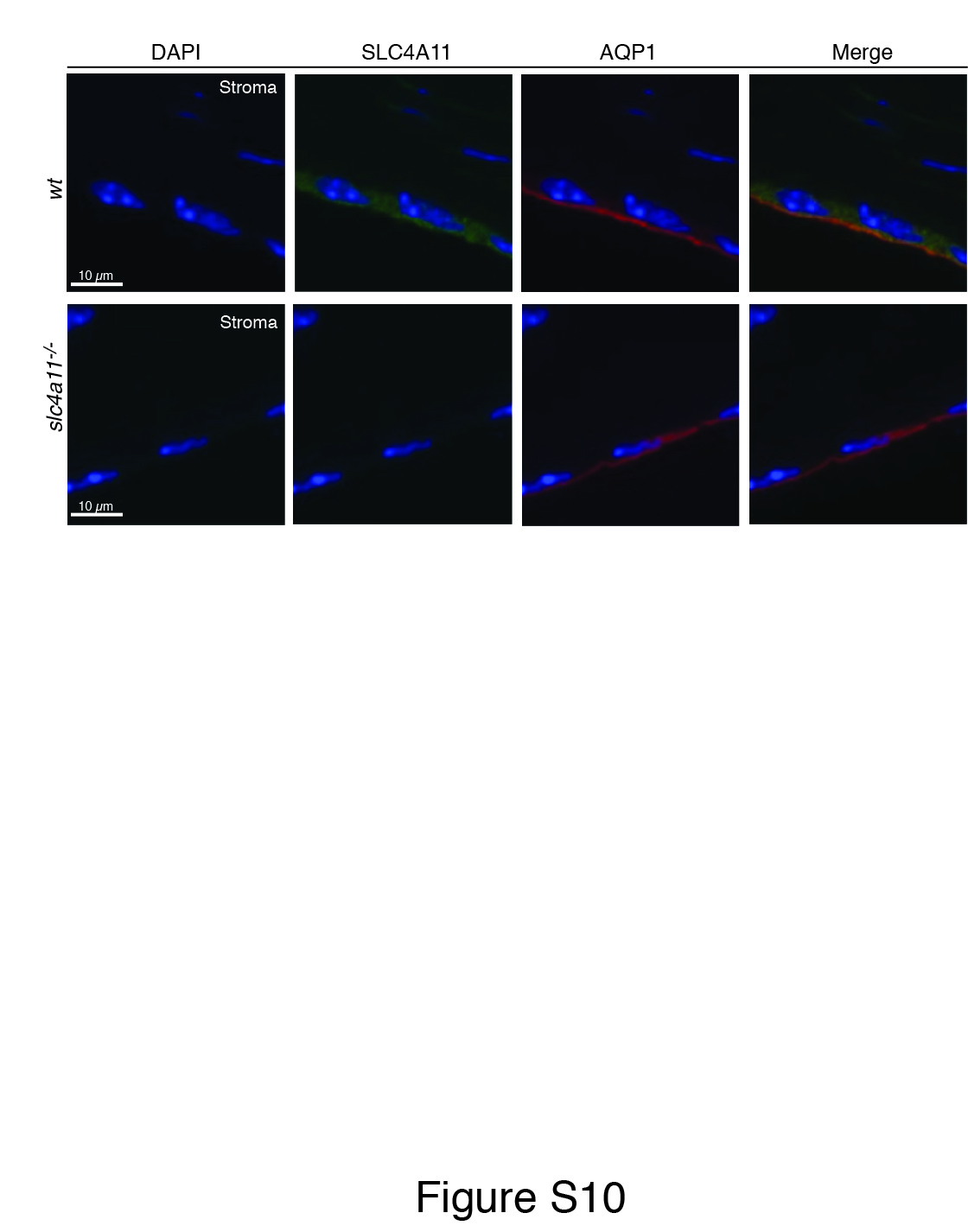


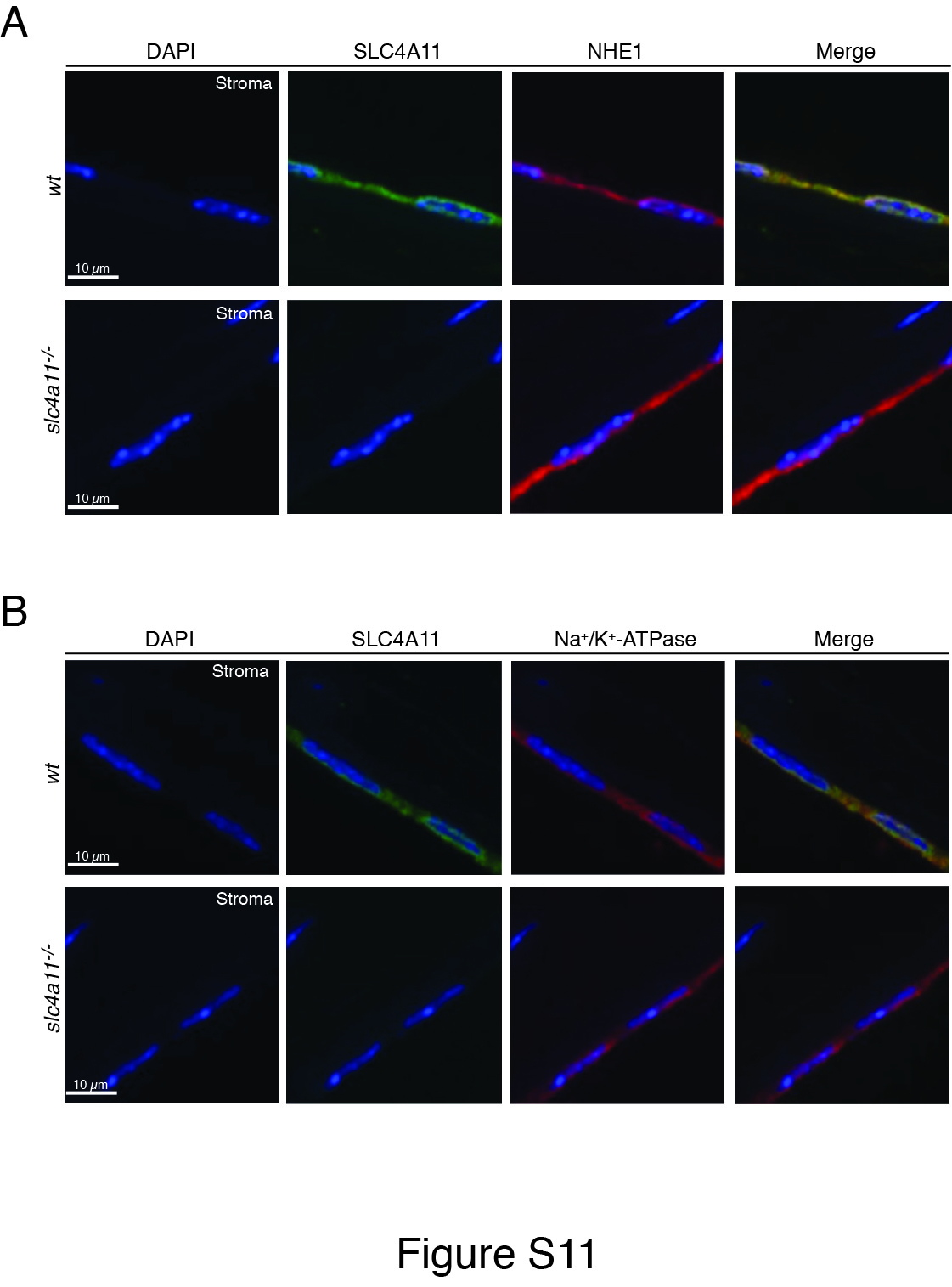

Supplement: Supplementary Data [file supp_ddt307_ddt307supp.doc]
